# Reorganization of the heterochromatin-associated gene-dense subcompartment in early neuronal development

**DOI:** 10.1242/bio.062005

**Published:** 2025-05-12

**Authors:** Nicolas J. Scrutton Alvarado, Ziyu Zhao, Tomoko Yamada, Yue Yang

**Affiliations:** ^1^ Department of Neurobiology, Northwestern University, Evanston, IL 60208, USA; ^2^ Program in Interdisciplinary Biological Sciences, Northwestern University, Evanston, IL 60208, USA

**Keywords:** Cerebellar granule neurons, Genome organization, Genomic subcompartment, Nuclear bodies, Doublecortin

## Abstract

The 3D organization of the genome has emerged as an important regulator of cellular development. Post-mitotic neurons undergo conserved changes in genome organization, such as the inward radial repositioning of heterochromatin-rich chromosomes as they differentiate. Additionally, transcriptionally active but heterochromatin-associated gene-dense (hGD) regions significantly strengthen their long-distance interactions during cerebellar development. However, the specific developmental stages during which these nuclear changes take place have remained poorly defined. Here, we report that hGD regions relocalize toward the nuclear interior and strengthen their chromosomal interactions as immature granule neurons transition from active cell migration to subsequent stages of neuronal differentiation. During this period, hGD genomic regions are coordinately repositioned in the nucleus alongside their physically tethered heterochromatic chromocenters. Despite these major changes in nuclear organization, the hGD subcompartment remains distinct from other transcriptionally active or repressive nuclear bodies, including heterochromatic chromocenters, throughout development. Notably, these nuclear changes appear to be independent of transcriptional changes that occur during granule neuron differentiation. Together, our results provide insights into the developmental timing of structural changes in the chromosomes of post-mitotic neurons.

## Teaser

**Summary:** Chromosomal reorganization of a transcriptionally active gene-dense subcompartment tethered to heterochromatic chromocenters occurs during early granule neuron development in the cerebellum.

## INTRODUCTION

The 3D organization of the genome is dynamically regulated alongside cellular development. This reorganization occurs across different genomic scales, spanning up to entire chromosomes (Fujita et al., 2022). Locally, gene promoters interact with regulatory enhancer elements to directly activate transcription. Beyond these short-range interactions, genomic regions organize at the chromosomal level into euchromatic and heterochromatic compartments, which can be further separated into smaller subcompartments (Rao et al., 2014). These subcompartments occupy distinct subnuclear locales and may be associated with nuclear bodies, often across different chromosomes (Quinodoz et al., 2018). The interaction of genomic loci with nuclear bodies, such as nuclear speckles, may enhance transcriptional or co-transcriptional processes, while genomic interaction with the nuclear lamina maintains gene repression in cells (Bhat et al., 2024; Reddy et al., 2008; Sabari et al., 2018). Thus, regulation of long-range genome organization provides additional mechanisms for controlling gene expression during development.

Post-mitotic neurons located throughout the mammalian brain have been found to undergo a set of seemingly common genome architectural changes, such as the radial repositioning of specific chromosomes. During the differentiation of olfactory sensory neurons, genomic regions containing olfactory receptor gene clusters may be relocated to a large heterochromatin cluster in the nuclear interior (Clowney et al., 2012). This is thought to ensure the constitutive repression of most olfactory receptor genes, while a single olfactory receptor gene may be chosen to escape the heterochromatin cluster and be expressed (Monahan et al., 2019). Similarly, large parts of chromosomes 7, 17, and 19 are repositioned toward the nuclear interior in cortical and hippocampal neurons during development (Takei et al., 2021; Tan et al., 2021; Winick-Ng et al., 2021). These regions are enriched for clustered gene families, such as vomeronasal receptors and immunoglobulins, which are constitutively repressed in mature neurons in the forebrain (Tan et al., 2021). These observations have been also extended to cerebellar granule neurons, the most abundant neuronal cell type in the brain (Tan et al., 2023; Zhao et al., 2023 preprint).

In the mouse cerebellum, we recently found that dense stretches of transcriptionally active genes, which are linearly adjacent to heterochromatin-silenced clustered gene families, robustly interacted with each other over long genomic distances to form a distinct genomic subcompartment in granule neurons (Zhao et al., 2023 preprint). This gene-dense subcompartment occupied a distinct microenvironment within the nucleus that is spatially separated from nuclear speckles and their associated genomic loci (Zhao et al., 2023 preprint). Remarkably, regions in the gene-dense subcompartment further strengthened their interactions in granule neurons during cerebellar development. However, we currently lack a detailed understanding of the specific stages of neuronal development during which these nuclear changes occur. Moreover, it remains unknown whether the transcriptionally active gene-dense subcompartment and linearly adjacent heterochromatin regions might be coordinately restructured during development.

Granule neurons offer a powerful system to study the mechanisms that regulate well-defined stages in neuronal development (Alvarez-Saavedra et al., 2014; Frank et al., 2015; Konishi et al., 2004; Okazawa et al., 2009; Yang et al., 2016). Granule neuron precursors expressing the excitatory neuron lineage marker Pax6 and mitotic cell marker Ki67 proliferate in the external granule layer (EGL) of the cerebellar cortex throughout the first to third postnatal weeks (Engelkamp et al., 1999; Shiraishi et al., 2019). During this period, waves of post-mitotic granule neurons are generated and express the microtubule-associated protein doublecortin (DCX), which is required for their migration through the EGL, molecular layer (ML), and Purkinje cell layer (PCL), before reaching their final location within the internal granule layer (IGL) (Consalez et al., 2021; Gleeson et al., 1999; Komuro and Rakic, 1998). There, granule neurons downregulate DCX expression as they integrate in cerebellar circuits by establishing connections with presynaptic and postsynaptic partners (Gleeson et al., 1999; Sepp et al., 2024; Weyer and Schilling, 2003). Thus, each of these developmental stages can be distinguished by the localization of granule neurons within cerebellar cortical layers and the use of validated protein markers, which together can be employed to elucidate developmental changes in genome organization.

In this study, we combined single-cell imaging with high-throughput genome profiling to identify the developmental periods for genome architectural changes in mouse cerebellar granule neurons. We focused our analyses on our recently identified gene-dense regions, which we collectively name the heterochromatin-associated gene-dense (hGD) subcompartment. We observed that the hGD subcompartment undergoes significant changes in chromosomal organization in post-mitotic neurons. Utilizing DNA fluorescence *in situ* hybridization combined with immunohistochemistry, we found that the radial repositioning and the strengthening of long-distance interactions between hGD regions occurred alongside the downregulation of DCX expression in granule neurons as they completed their migration within the IGL. We also discovered, using fluorescence-activated nuclei sorting (FANS) followed by Hi-C analyses, that the entire hGD subcompartment underwent chromosomal reorganization during early granule neuron development. The timing of these major changes in the hGD subcompartment coincided with the nuclear repositioning of heterochromatic chromocenters tethered to hGD regions, though these adjacent structures maintained distinct spatial compartments throughout development. Unexpectedly, we also found that the strengthening of hGD subcompartment interactions was independent of developmental changes in gene expression within these regions. Together, our results reveal the developmental window for the structural rearrangements of chromosomes that are decoupled from transcriptional regulation in granule neurons.

## RESULTS

### hGD regions are inwardly repositioned over cerebellar granule neuron development

**Fig. 1.**
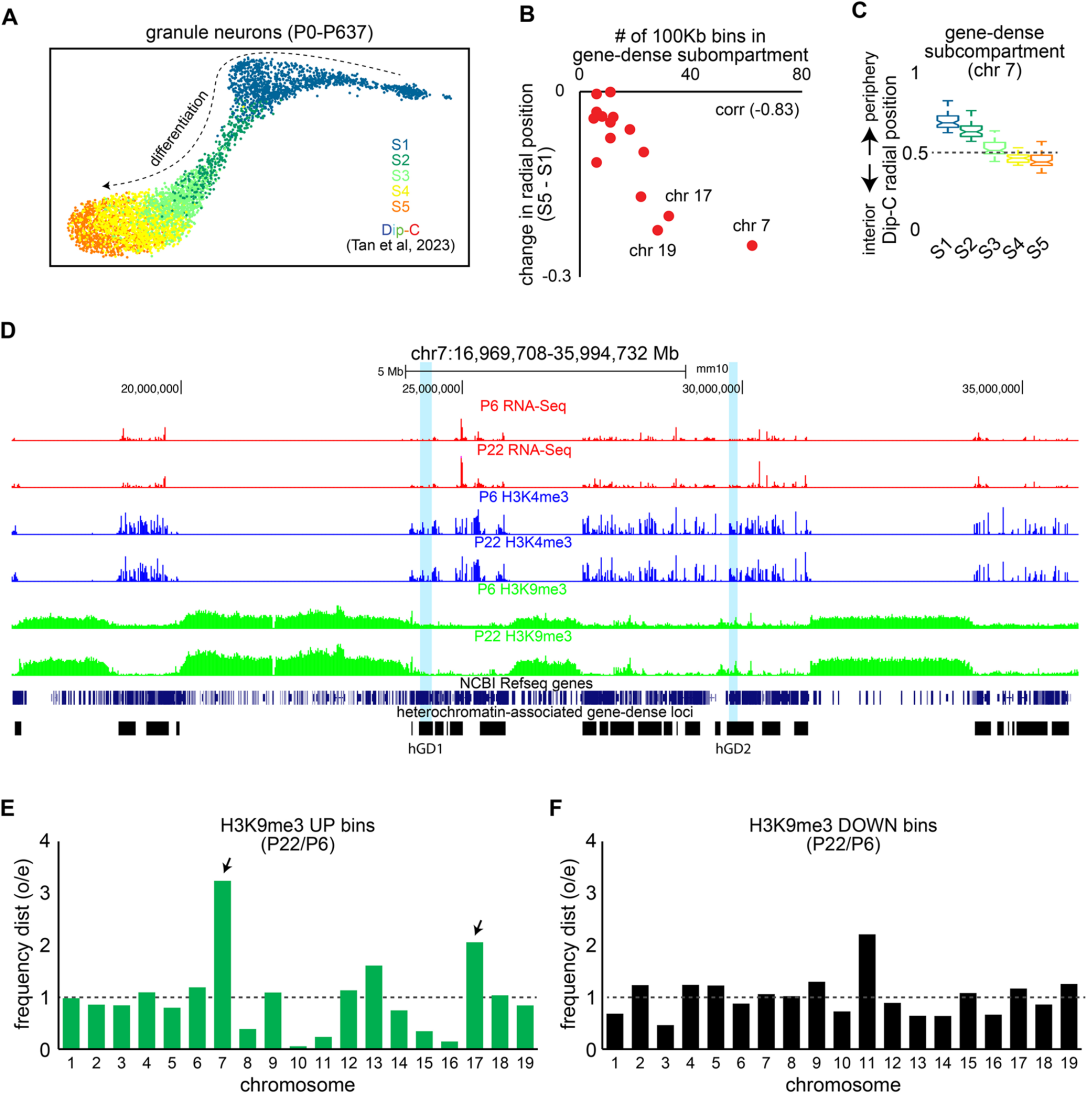
**hGD regions are repositioned toward the nuclear interior over granule neuron development.** (A) UMAP plots of cerebellar granule neurons from postnatal day 0 (P0) to P637 mice subjected to Dip-C analyses (Tan et al., 2023). The genome structural stages (S1-S5) identified in granule neurons, which reflect their differentiation, are shown. (B) Changes in radial positioning of gene-dense subcompartment loci at 100Kb resolution across chromosomes between structural stages S1 to S5. Chromosome 7 contained the most gene-dense subcompartment regions and these regions exhibited the largest inward radial changes across structural stages S1 and S5. These two features were also correlated across chromosomes. (C) Changes in the radial position of gene-dense subcompartment regions on chromosome 7 over granule neuron structural stages. Radial repositioning of these regions occurred mostly between S1 to S3. (D) UCSC genome browser tracks showing gene-dense subcompartment regions (black) and NCBI Refseq genes (dark blue) on chromosome 7 alongside RNA-seq (red), H3K4me3 (blue), and H3K9me3 (green) levels in the cerebellum at postnatal day 6 (P6), when granule neuron precursors and immature post-mitotic granule neurons are most abundant, and at postnatal day 22 (P22), when most cells are mature granule neurons. DNA FISH probes targeting the heterochromatin-associated gene-dense (hGD) regions hGD1 and hGD2, located 5.6Mb apart, are highlighted in light blue. hGD1 spans 255 kb and hGD2 spans 480 kb. (E,F) The frequency distribution of genomic bins across chromosomes showing increased (E) or decreased (F) H3K9me3 levels in the cerebellum of P6 and P22 mice. Chromosomes 7 and 17 contained a high proportion of genomic regions with increased H3K9me3 levels during cerebellar development. In panel C, boxplots show median, first to third quartiles (boxes), and sample range (whiskers). Notches indicate confidence intervals.

We recently identified a gene-dense subcompartment that is distinct from other transcriptionally active nuclear subcompartments in the cerebellum (Zhao et al., 2023 preprint). This subcompartment is composed of several hundred genomic loci enriched on chromosomes 7 and 17, which preferentially associate within the nuclear interior (Zhao et al., 2023 preprint). To understand how this subcompartment is changed during development, we first analyzed 3D models of developing and adult cerebellar granule neurons from published single-cell diploid chromatin conformation capture (Dip-C) datasets (Fig. 1A) (Tan et al., 2023). Among chromosomes, gene-dense regions within chromosome 7 exhibited the most prominent inward radial movements as granule neurons differentiated (Fig. 1B). When grouping granule neurons by their Dip-C structural stage (S1-S5), inferred through unsupervised clustering (Tan et al., 2023), gene-dense subcompartment regions within chromosome 7 showed the most pronounced repositioning toward the nuclear interior during structural stages S1 to S3 (Fig. 1C).

Given the significant reorganization of the gene-dense subcompartment within chromosome 7 during granule neuron development, we examined the chromatin landscape surrounding these genomic regions to identify factors associated with the observed structural changes. As expected, gene-dense subcompartment regions were marked by histone H3 lysine 4 trimethylation (H3K4me3) and showed robust gene expression, suggesting that they are transcriptionally active (Fig. 1D). These regions were also found adjacent to broad heterochromatin domains marked by histone H3 lysine 9 trimethylation (H3K9me3) (Fig. 1D). We therefore reclassified these transcriptionally active genomic loci as hGD regions, based on their surrounding repressive chromatin environment. These hGD regions may be distinguished from nuclear speckle-associated genomic regions, which are also gene-dense but do not neighbor heterochromatin regions (Chen et al., 2018). Additionally, we observed that heterochromatin regions on chromosome 7 and 17 exhibited increased H3K9me3 levels during cerebellar development (Fig. 1E,F), a feature associated with the strengthening of heterochromatin compartmentalization in post-mitotic neurons (Bashkirova et al., 2023). Together, these results suggest that the nuclear reorganization of hGD regions may be associated with developmental changes in their adjacent heterochromatin domains in the cerebellum.

### hGD loci undergo radial repositioning and increase their 3D proximity during cerebellar development

**Fig. 2.**
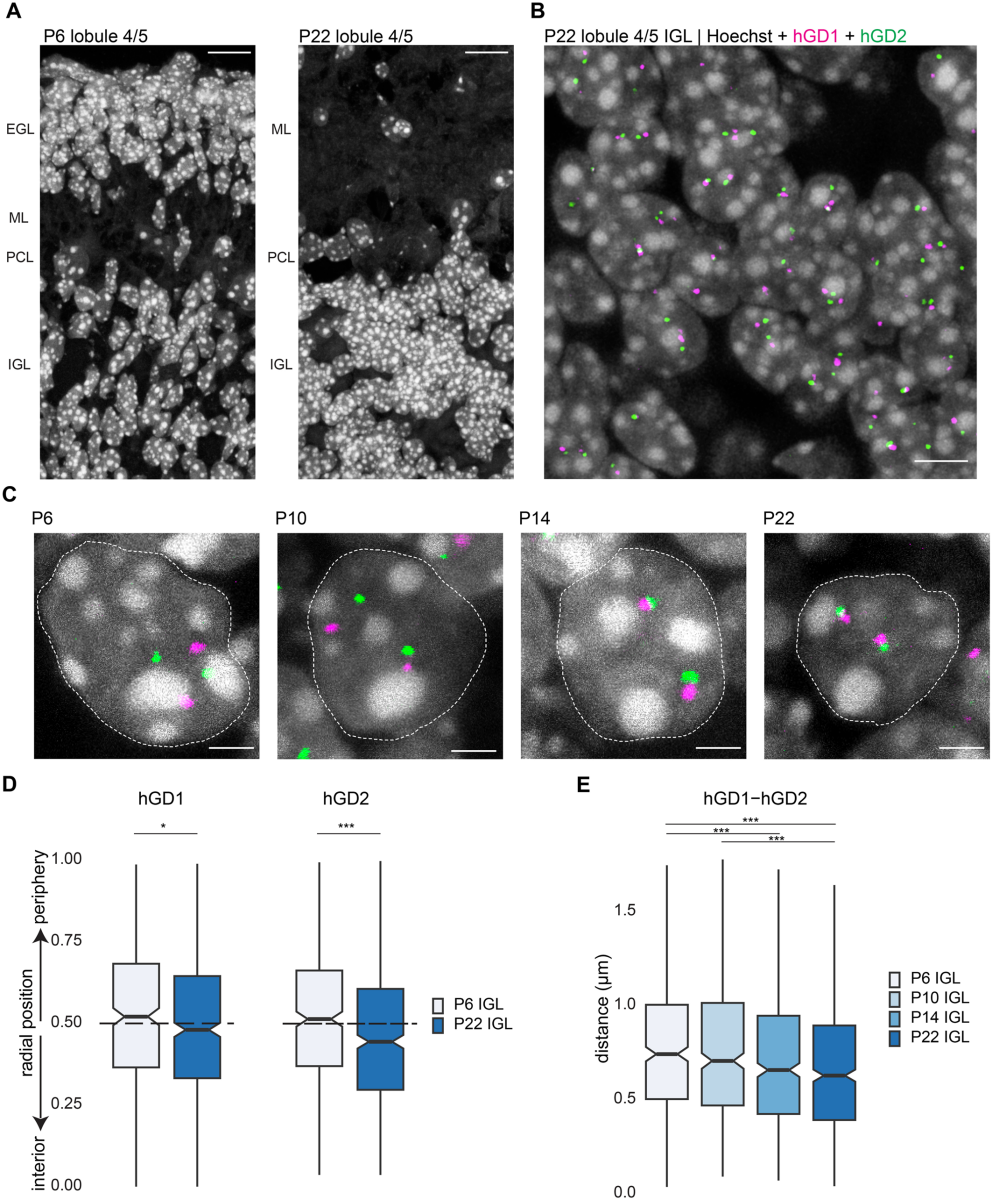
**hGD loci undergo changes in radial positioning and spatial proximity during cerebellar development.** (A) The cerebellar cortex in lobule 4/5 from P6 (left) or P22 mice (right) labeled with Hoechst DNA dye. EGL, external granule layer; ML, molecular layer; PCL, Purkinje cell layer; IGL, internal granule layer. Scale bars: 10 µm. (B) The IGL of cerebellar lobule 4/5 from P22 mice subjected to DNA FISH analyses using the hGD1 (magenta) and hGD2 (green) probes and stained with Hoechst dye (gray). Scale bar: 5 µm. (C) DNA FISH analyses using the P6, P10, P14, or P22 mouse cerebellum and prepared as in B. Images of granule neuron nuclei are shown, with the dashed line delineating the nuclear boundary. Scale bars: 2 µm. (D) The radial positioning of hGD1 and hGD2 loci using the cerebellum from P6 and P22 mice and prepared as in C. Both hGD loci were inwardly repositioned during development (*P<0.05, ***P<0.001 using two-sided Mann–Whitney-Wilcoxon rank sum test, *n*=361, 514 for hGD1 P6, P22, *n*=407, 553 for hGD2 P6, P22). (E) The 3D distances between hGD1 and hGD2 loci using the cerebellum from P6, P10, P14, and P22 mice and prepared as in C. hGD loci gradually increased their proximity across cerebellar development, with significant changes beginning at P14 (***P<0.001 using Kruskal–Wallis rank sum test with Dunn's post-hoc test, *n*=445, 445, 509, 625 for P6, P10, P14, P22). In panels D and E, boxplots show median, first to third quartiles (boxes), and sample range (whiskers). Notches represent confidence intervals of the median. All data were collected from two independent biological replicates.

The predictions from Dip-C 3D models that hGD regions change their nuclear localization in developing granule neurons led us to visualize these regions using 3D DNA fluorescence *in situ* hybridization (DNA FISH) in the cerebellum of postnatal mice. We generated probes targeting two hGD regions (hGD1 and hGD2) located 5.6Mb apart on chromosome 7 (Fig. 1D). We examined several cerebellar ages during development, including postnatal day 6 (P6), when the cerebellum is primarily composed of both granule neuron precursors in the external granule layer (EGL) and immature post-mitotic granule neurons in the internal granule layer (IGL), as well as postnatal day 22 (P22), when the EGL has disappeared and mature granule neurons in the IGL have integrated into cerebellar cortical circuits (Fig. 2A) (Yamada et al., 2014). When comparing granule neurons in the IGL at these developmental ages, we observed a significant shift of the hGD1 and hGD2 regions toward the nuclear interior during granule neuron differentiation (Fig. 2B-D).

Next, we asked whether these changes in the radial positioning of hGD regions were associated with other alterations in nuclear organization. Using Hi-C analyses, we have previously found that hGD regions showed increases in interaction frequency with cerebellar development (Zhao et al., 2023 preprint). Therefore, we reasoned that the re-localization of these regions closer to the nuclear interior might also enhance their spatial association in granule neurons during development. To visualize these changes, we examined the 3D spatial distance between the hGD1 and hGD2 regions using DNA FISH analyses of granule neurons in the IGL. We found that the 3D distance between the two hGD regions decreased with cerebellar age during development, with significant changes beginning at P14 and continuing through to P22 (Fig. 2E). Together, these results suggest that the inward radial positioning of hGD regions is accompanied by an increase in their spatial proximity during granule neuron differentiation.

### hGD nuclear reorganization is associated with DCX downregulation in post-migratory granule neurons

**Fig. 3.**
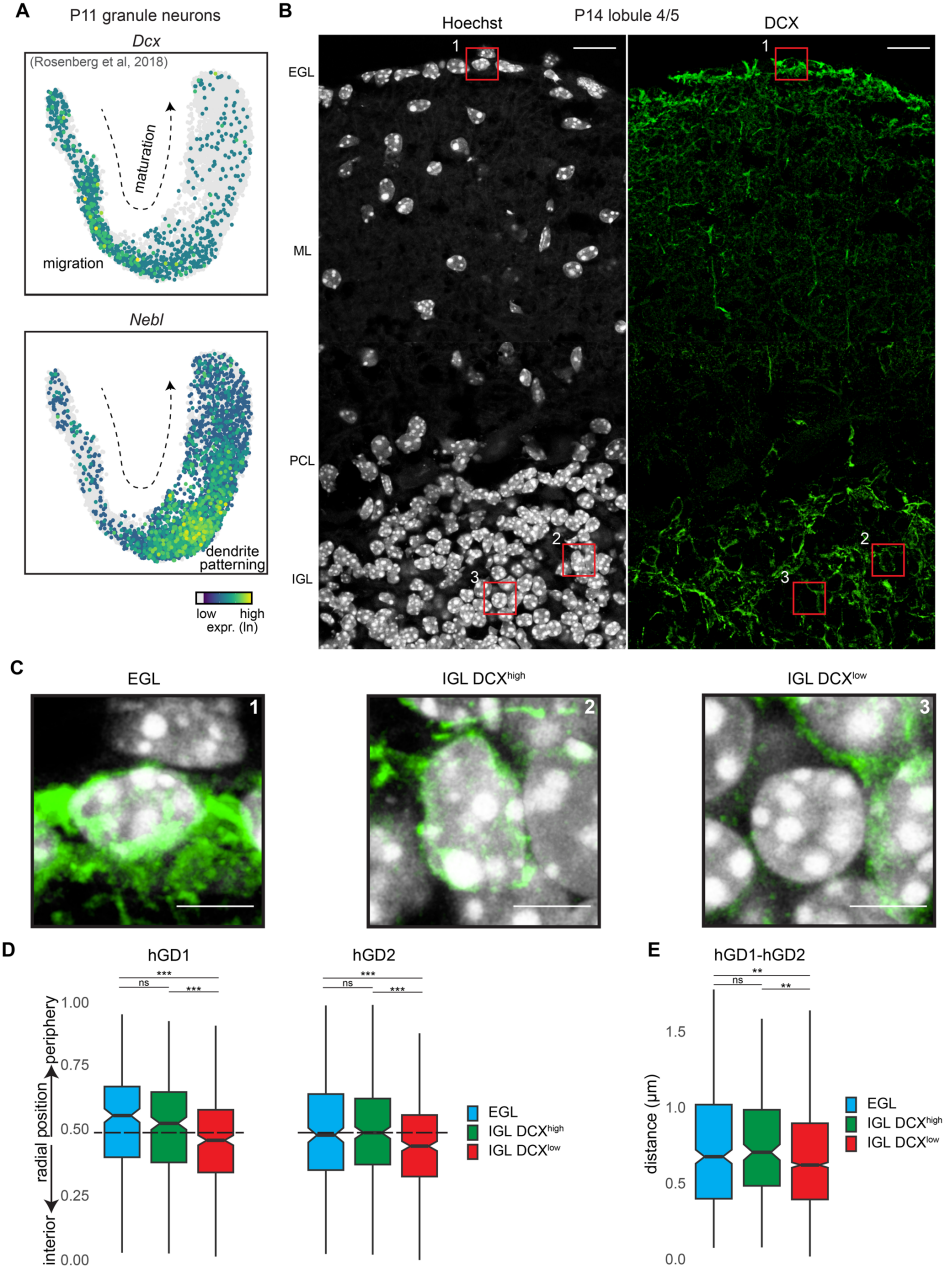
**hGD reorganization is associated with DCX downregulation in post-migratory granule neurons.** (A) *Dcx* (top) or *Nebl* (bottom) expression visualized using a UMAP plot of snRNA-seq data from developing cerebellar granule neurons in P11 mice (Rosenberg et al., 2018). High *Dcx* expression marked immature post-mitotic granule neurons undergoing cell migration, while high *Nebl* expression marked a post-migratory developmental stage. (B) Immunohistochemical analyses of cerebellar sections in lobule 4/5 prepared from P14 mice labeled with Hoechst dye (left) and an antibody against DCX (right). Scale bars: 20 µm. (C) Three regions from B (red squares) depicting immature granule neurons in the EGL, along with those showing high DCX expression (DCX^high^) or low DCX expression (DCX^low^) in the IGL. Scale bars: 5 µm. (D) The radial positioning of hGD1 and hGD2 loci in granule neurons from the developmental stages shown in C and Fig. S1A. Both hGD loci relocated toward the nuclear interior in DCX^low^ post-migratory granule neurons (**P<0.01, ***P<0.001 using Kruskal–Wallis rank sum test with Dunn's post-hoc test, *n*=263, 304, 393 for hGD1 EGL, IGL DCX^high^, IGL DCX^low^, *n*=273, 310, 397 for hGD2 EGL, IGL DCX^high^, IGL DCX^low^). (E) The distances between hGD1 and hGD2 loci across the developmental stages shown in C and Fig. S1A. hGD loci increased in proximity in DCX^low^ post-migratory granule neurons compared to earlier development stages (**P<0.01 using Kruskal–Wallis rank sum test with Dunn's post-hoc test, *n*=188, 138, 1458 for EGL, IGL DCX^high^, IGL DCX^low^). In panels D and E, boxplots show median, first to third quartiles (boxes), and sample range (whiskers). Notches represent confidence intervals of the median. All data were collected from two independent biological replicates.

The observed changes in both radial positioning and spatial proximity of hGD regions over development led us to determine whether these rearrangements occurred at specific stages of granule neuron differentiation. Given the heterogeneity of granule neurons in the early postnatal cerebellum, we used markers to identify granule neuron subpopulations at distinct developmental stages. Single nuclei (sn)RNA-seq analyses of cerebellar granule neurons in P11 mice (Rosenberg et al., 2018) revealed genes expressed at defined developmental stages, such as *Dcx*, which encodes a microtubule-associated protein enriched in immature post-mitotic granule neurons undergoing migration, and *Nebl*, which encodes an actin-binding protein transiently expressed at a later developmental stage in post-migratory granule neurons (Fig. 3A) (Gleeson et al., 1999; Myers et al., 2020). Notably, in the cerebellum of P14 mice, newly-born post-mitotic granule neurons undergoing cell migration from the EGL toward the IGL possessed a distinctive ring of DCX protein fluorescence around their nuclei (Fig. 3B,C). This DCX ring was then lost, likely as neurons completed their migration and settle in their final position in the IGL (Fig. 3B,C) (Gleeson et al., 1999; Huynh et al., 2011; Zimatkin and Karnyushko, 2017).

To examine how the nuclear positioning of hGD regions was changed at various granule neuron developmental stages, we performed DCX immunolabeling combined with DNA FISH analyses. We observed no significant differences in the radial positioning of hGD1 and hGD2 regions in DCX-expressing granule neurons that are likely migrating in the cerebellar cortex, including in post-mitotic neurons in the inner EGL and a subset of neurons within the IGL with high DCX expression (IGL DCX^high^) (Fig. 3D, Fig. S1). Strikingly, however, we found a significant shift of both hGD1 and hGD2 regions toward the nuclear interior in IGL granule neurons with little or no DCX expression (IGL DCX^low^) (Fig. 3D, Fig. S1). In conjunction with these changes, we also observed a reduction in the 3D distance between the hGD1 and hGD2 regions in post-migratory granule neurons with low DCX expression, compared to earlier stages in migrating granule neurons with high DCX expression (Fig. 3E). This genome reorganization occurred without changes in nuclear area between DCX^high^ and DCX^low^ granule neurons, although changes were observed with cerebellar age (Fig. S2). Together, these results suggest that changes in the nuclear organization of hGD regions, including their radial repositioning and spatial proximity, occur as granule neurons switch from a phase of cell migration to later stages when they integrate in cerebellar circuits.

### hGD regions are radially repositioned alongside chromocenters toward the nuclear interior

**Fig. 4.**
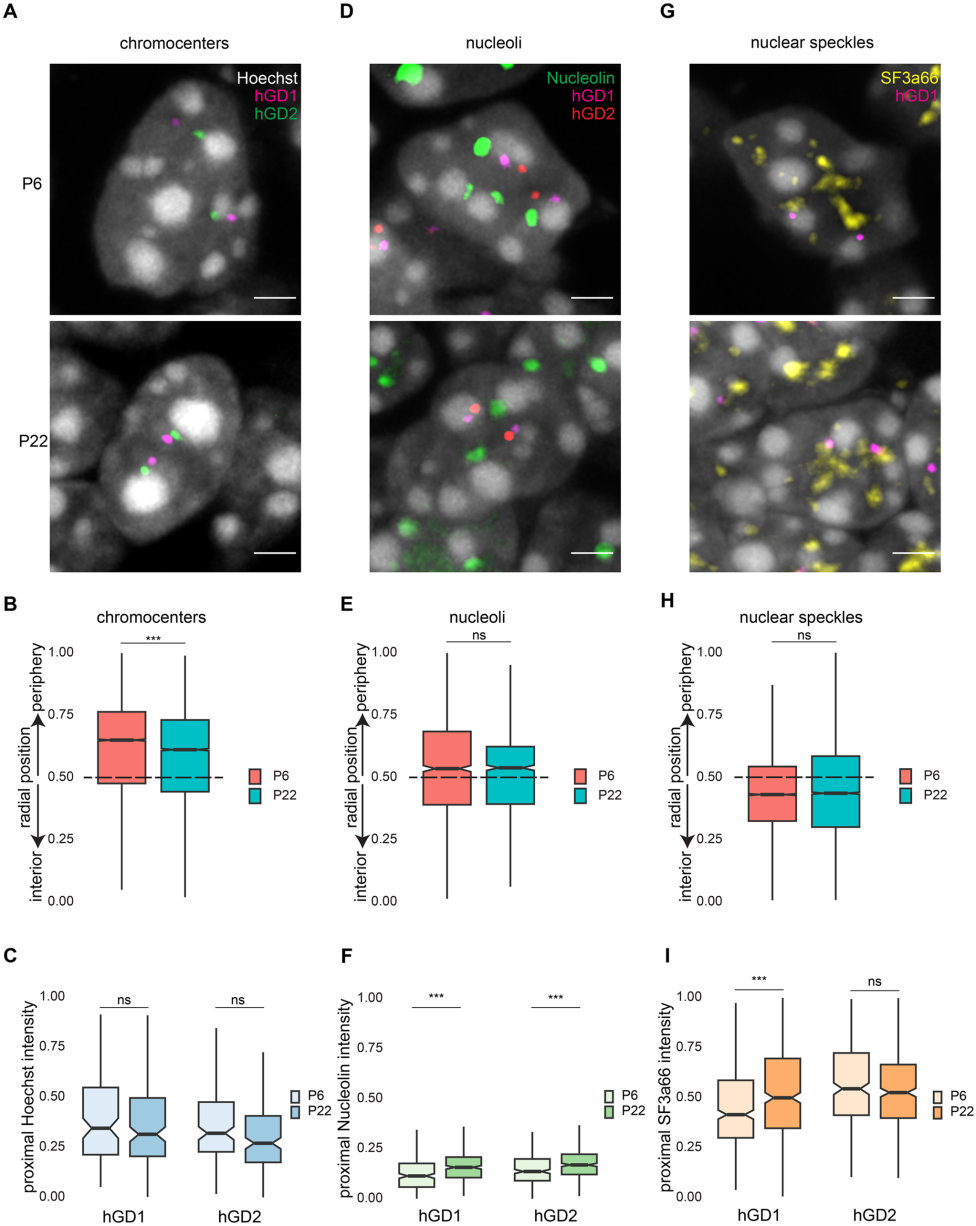
**hGD regions are radially repositioned alongside chromocenters but they remain spatially separated.** (A) Granule neuron nuclei in cerebellar lobule 4/5 from P6 (top) or P22 mice (bottom) labeled with Hoechst dye (gray) to visualize chromocenters densely packed with DNA, together with the hGD1 (magenta) and hGD2 (green) DNA FISH probes. Scale bars: 2 µm. (B) The radial positioning of chromocenters as shown in A. Chromocenters were repositioned away from the outermost nuclear periphery during development (***P<0.001 using Asymptotic Two-Sample Brown-Mood Median Test, *n*=10,879, 13,923 for P6, P22). (C) Normalized Hoechst fluorescence intensity within a 350 nm radius circle surrounding hGD loci in granule neurons from P6 or P22 cerebellum. Little or no changes in Hoechst fluorescence levels around hGD loci were observed over cerebellar development (*P*=0.1542, *P*=0.1115 using Asymptotic Two-Sample Brown-Mood Median Test, *n*=122, 168 for hGD1 P6, P22, *n*=154, 177 for hGD2 P6, P22). (D) Granule neuron nuclei in cerebellar lobule 4/5 from P6 (top) or P22 mice (bottom) labeled with Hoechst dye (gray), an antibody against Nucleolin (green) to visualize nucleoli, and the hGD1 (magenta) and hGD2 (red) DNA FISH probes. Scale bars: 2 µm. (E) The radial positioning of nucleoli as shown in D. Nucleoli were positioned close to the nuclear midline, with no little or no change across development (*P*=0.8434 using Asymptotic Two-Sample Brown-Mood Median Test, *n*=1389, 1187 for P6, P22). (F) Normalized Nucleolin immunofluorescence intensity analyzed as in C using P6 or P22 cerebellum. Nucleolin immunofluorescence levels around hGD loci showed modest increases during development (***P<0.001 using Asymptotic Two-Sample Brown-Mood Median Test, for hGD1 P6, P22, *n*=276, 377 for hGD2 P6, P22). (G) Granule neuron nuclei in cerebellar lobule 4/5 from P6 (top) or P22 mice (bottom) labeled with Hoechst dye (gray), an antibody against SF3a66 (yellow) to visualize nuclear speckles, and the hGD1 (magenta) DNA FISH probe. Scale bars: 2 µm. (H) The radial positioning of nuclear speckles as shown in G. Nuclear speckles were positioned in the nuclear interior, showing little or no overall movement across development (*P*=0.06775 using Asymptotic Two-Sample Brown-Mood Median Test, *n*=12,067, 11,270 for P6, P22). (I) Normalized SF3a66 immunofluorescence intensity analyzed as in C using P6 or P22 cerebellum. SF3a66 immunofluorescence levels around the hGD1 locus increased during cerebellar development (***P<0.001, *P*=0.1828 using Asymptotic Two-Sample Brown-Mood Median Test, *n*=446, 454 for hGD1 P6, P22, *n*=522, 592 for hGD2 P6, P22), reaching levels comparable to those around the hGD2 locus. In panels B,C,E,F,H, and I, boxplots show median, first to third quartiles (boxes), and sample range (whiskers). Notches represent confidence intervals of the median. All data were collected from two independent biological replicates.

We next asked whether these genome architectural changes might be linked to changes in association with nuclear bodies such as chromocenters, nucleoli, or nuclear speckles, which have roles in organizing chromosomal hubs in cells (Quinodoz et al., 2018). Since hGD regions are linearly adjacent to large heterochromatin domains on the genome, we reasoned that they might also be physically positioned nearby heterochromatic chromocenters in the nucleus_­_. We first examined the localization of chromocenters, which may be visualized using the Hoechst DNA dye, in granule neurons. We found that initially in P6 mice, chromocenters were positioned nearby the nuclear lamina at the nuclear periphery in immature granule neurons (Fig. 4A,B). However, in mature granule neurons in P22 mice, heterochromatic chromocenters shifted modestly away from the periphery, possibly as a result of heterochromatin regions undergoing radial repositioning (Fig. 4A,B). Indeed, we observed that H3K9me3-marked heterochromatin regions (H1 and H2) adjacent to hGD1/2 regions within chromosome 7 were robustly repositioned toward the nuclear interior during neuronal differentiation (Fig. S3A-C). Moreover, similar to hGD regions, H1 and H2 also exhibited a significant decrease in 3D distance over cerebellar development (Fig. S3D). To determine the spatial relationship between transcriptionally active hGD regions and heterochromatic chromocenters, we next measured the enrichment of chromocenters within a 350 nm radius circle surrounding the hGD probes. We found that throughout granule neuron development, there was a weaker overlap between chromocenters and the hGD1 and hGD2 regions compared to chromocenters and the hGD-adjacent heterochromatin regions H1 and H2 (Fig. 4C, Fig. S3E). Together, these data suggest that hGD regions and their physically tethered heterochromatic chromocenters are both repositioned away from the nuclear periphery during granule neuron differentiation, but these structures retained 3D spatial separation throughout development.

In addition to the coordinated radial repositioning of chromocenters and hGD regions, we investigated whether hGD regions might be associated with other nuclear bodies. Nucleolin-marked nucleoli were found at the periphery of chromocenters (Fig. 4D), where they may establish a nucleolar-associated inter-chromosomal hub (Quinodoz et al., 2018). As observed for hGD regions, nucleoli were positioned at an intermediate radial location between the nuclear periphery and nuclear interior (Fig. 4D,E). However, unlike hGD regions, nucleoli positioning remained largely unchanged throughout cerebellar development (Fig. 4E). We also found that the association between hGD regions and nucleoli was substantially lower compared to the association of hGD regions with chromocenters during development (Fig. 4C,F). These findings suggest that changes in the organization of hGD regions with granule neuron differentiation is largely independent of nucleolar association in granule neurons.

Besides heterochromatin-associated structures, we also characterized nuclear speckles, which are hubs for splicing factors that boost the mRNA processing of proximal gene loci (Bhat et al., 2024; Sung et al., 2023). Like hGD regions, nuclear speckle-associated genomic regions contain dense stretches of genes and are sites of high levels of mRNA transcription (Quinodoz et al., 2018). We observed that nuclear speckles, marked by the splicing factor SF3a66, were primarily located in the nuclear interior throughout granule neuron development and were well separated from heterochromatic chromocenters (Fig. 4G,H, Fig. S4A). Similar observations on nuclear positioning were made for a genomic region (NS1) that is robustly associated with nuclear speckles in a conserved manner from embryonic stem cells to granule neurons (Fig. S4B,C) (Quinodoz et al., 2018; Zhao et al., 2023 preprint). When examining the association of hGD regions with nuclear speckles, we observed a modest enrichment of the nuclear speckle marker SF3a66 around the hGD2 region compared to the hGD1 region in immature granule neurons at P6 (Fig. 4I). With development, the hGD1 region increased its association with nuclear speckles, matching that of the hGD2 region in mature granule neurons at P22, although both hGD regions remained weaker than that of the NS1 genomic region (Fig. 4I, Fig. S4B). Consistent with these findings, the 3D distance between hGD1 and the nearest nuclear speckle border significantly decreased between P6 and P22, while the NS1 locus maintained a closer proximity to nuclear speckles even at the later stage (Fig. 4I, Fig. S4E). Together, our findings suggest that during granule neuron differentiation, hGD regions move in concert with their tethered chromocenters from the nuclear periphery toward the interior, where nuclear speckles reside. However, throughout development, these hGD regions maintain partial spatial separation from other nuclear bodies over a distance of a few hundred nanometers, which may reflect their distinct spatial compartmentalization.

### Nuclear markers capture distinct granule neuron developmental stages for biochemical analysis

**Fig. 5.**
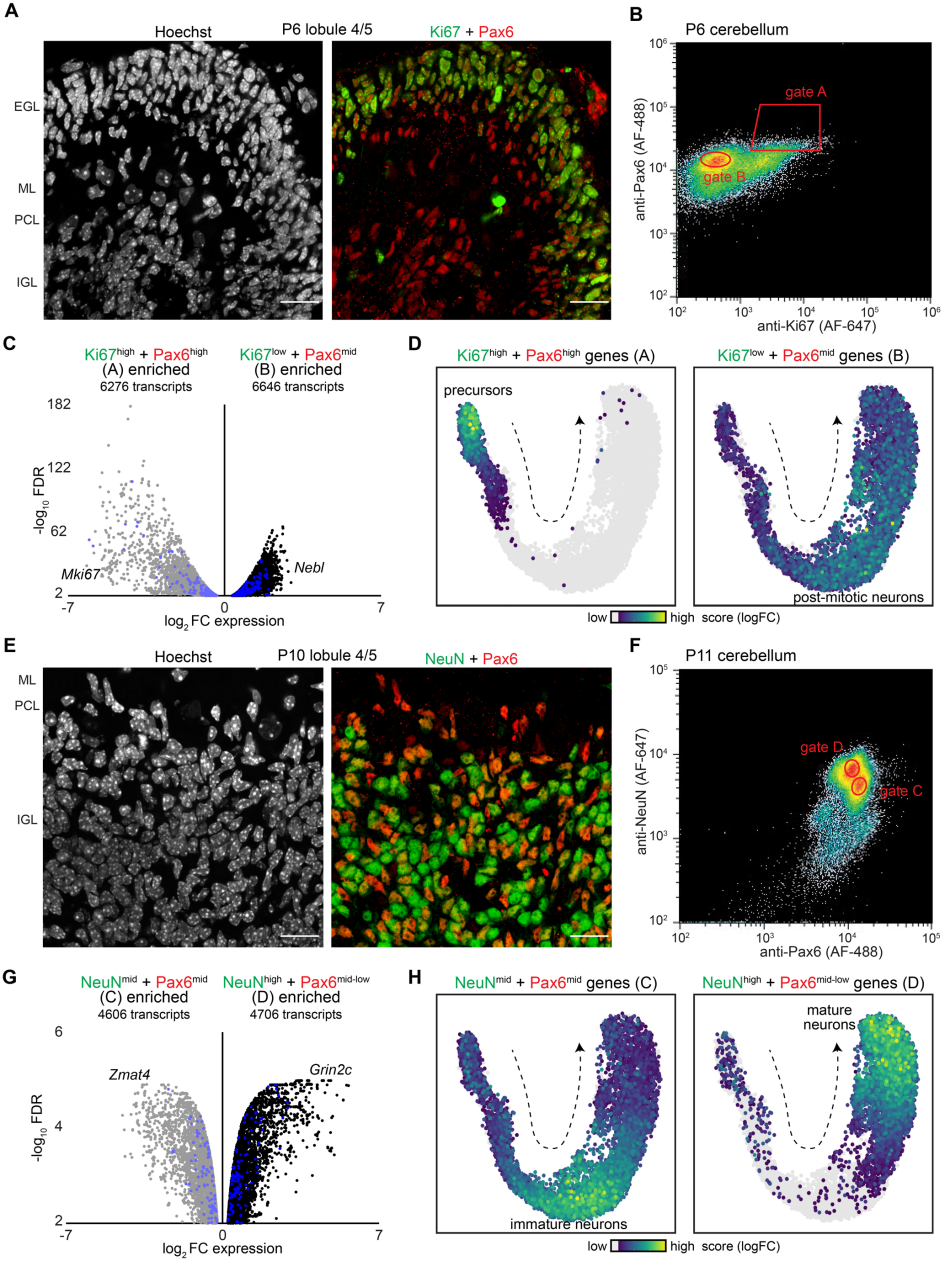
**Isolation of granule neurons at distinct developmental stages using nuclear markers.** (A) The cerebellar cortex in lobule 4/5 from a P6 mouse labeled with antibodies against Ki67 (green) and Pax6 (red) together with Hoechst dye (gray). Pax6 selectively marked the granule neuron lineage, while Ki67 marked mitotic granule neuron precursors in the outer EGL but was absent in immature post-mitotic granule neurons in the inner EGL and IGL. Scale bars: 20 µm. (B) Dissociated nuclei from the cerebellum of P6 mice were labeled with antibodies against Ki67 and Pax6 and subjected to fluorescence-activated nuclei sorting (FANS). The gates A and B were used to isolate two granule neuron populations for downstream biochemical analyses. (C) A volcano plot showing enriched transcripts in the P6 FANS-isolated populations from B, including *Mki67* in Ki67^high^/Pax6^high^ granule neurons and *Nebl* in Ki67^low^/Pax6^mid^ granule neurons. Transcripts found within hGD regions are highlighted in blue. (D) Gene modules that define Ki67^high^/Pax6^high^ (left) or Ki67^low^/Pax6^mid^ (right) granule neurons were visualized using a UMAP plot of snRNA-seq data as in Fig. 3A. Genes enriched in the Ki67^high^/Pax6^high^ population marked granule neuron precursors on the UMAP plot, while genes enriched in the Ki67^low^/Pax6^mid^ population marked immature post-mitotic granule neurons. (E) The cerebellar cortex in lobule 4/5 of a P10 mouse labeled with antibodies against NeuN (green) and Pax6 (red) together with Hoechst dye (gray). The relative levels of NeuN and Pax6 distinguished granule neuron populations within the IGL. Scale bars: 20 µm. (F) Dissociated nuclei from the cerebellum of P11 mice were labeled with antibodies against Pax6 and NeuN and subjected to FANS. The gates C and D were used to isolate two discernable granule neuron populations for downstream analyses. (G) A volcano plot showing enriched transcripts in the P11 FANS-isolated populations from F, including *Zmat4* in NeuN^mid^/Pax6^mid^ granule neurons and *Grin2c* in NeuN^high^/Pax6^mid-low^ granule neurons. Transcripts located within hGD regions are highlighted in blue. (H) Gene modules that define NeuN^mid^/Pax6^mid^ (left) or NeuN^high^/Pax6^mid-low^ (right) granule neurons were visualized with UMAP as in Fig. 3A. Genes enriched in the NeuN^mid^/Pax6^mid^ population marked immature post-mitotic granule neurons on the UMAP plot, while genes enriched in the NeuN^high^/Pax6^mid-low^ population marked mature granule neurons.

Given our observation that individual hGD regions displayed significant nuclear changes during granule neuron development using microscopy, we next determined whether the 3D organization of the entire hGD subcompartment, including their genomic interactions across chromosomes, might be similarly altered. We therefore sought to take advantage of biochemical approaches such as Hi-C, which can map chromosomal organization with genome-wide resolution, to study granule neurons at discrete stages of their development. We first identified suitable protein markers for isolating the nuclei from subpopulations of developing granule neurons for downstream biochemical analyses. Well established nuclear markers include Pax6, which is expressed selectively in the excitatory granule neuron lineage, and Ki67, which labels actively proliferating cells (Engelkamp et al., 1999; Weisheit et al., 2006). In immunohistochemical analyses of the cerebellar cortex from P6 mice, we observed granule neuron precursors expressing high levels of Ki67 and Pax6 throughout the outer EGL (Fig. 5A). By contrast, immature post-mitotic granule neurons lacking Ki67 expression but expressing moderate levels of Pax6 were found predominantly in the inner EGL and the nascent IGL (Fig. 5A). We next used FANS to isolate the nuclei of granule neuron precursors and immature post-mitotic granule neurons by using the Ki67 and Pax6 markers in P6 mice (Fig. 5B). In RNA-seq analyses of the Ki67^high^/Pax6^high^ and Ki67^low^/Pax6^mid^ FANS-isolated populations, we identified 6759 differentially expressed genes, including *Mki67* mRNA, which is enriched in granule neuron precursors, and *Nebl* mRNA, which is enriched in immature post-mitotic granule neurons (Figs 3A and 5C). The top genes enriched in the two populations can be visualized on a snRNA-seq UMAP projection of developing granule neurons, which includes both granule neuron precursors and immature post-mitotic granule neurons (Fig. 5D) (Rosenberg et al., 2018).

Besides enriching for granule neuron populations at earlier developmental stages, we also sought to isolate granule neurons at later stages, as these neurons integrate into cerebellar circuits. In immunohistochemical analyses of the cerebellar cortex of P10 mice, we visualized granule neurons using the protein markers Pax6, which is partially downregulated as neurons mature, and the splicing factor NeuN, encoded by the *Rbfox3* gene, which is upregulated throughout neuron differentiation (Fig. 5E). Importantly, the relative abundance of the Pax6 and NeuN markers distinguished at least two major granule neuron populations in the IGL, as observed using FANS and immunohistochemical analyses of P10-P11 mice (Fig. 5E,F, Fig. S5A,B). The Pax6^mid^ and Pax6^mid-low^ granule neuron populations exhibited low DCX protein levels (Fig. S5C,D), suggesting that these neurons are post-migratory. RNA-seq analyses of the NeuN^mid^/Pax6^mid^ and NeuN^high^/Pax6^mid-low^ populations revealed distinct transcriptional signatures, which mapped onto immature and mature post-mitotic granule neuron populations, respectively (Fig. 5G,H).

### The hGD subcompartment is strengthened during early post-mitotic granule neuron development

**Fig. 6.**
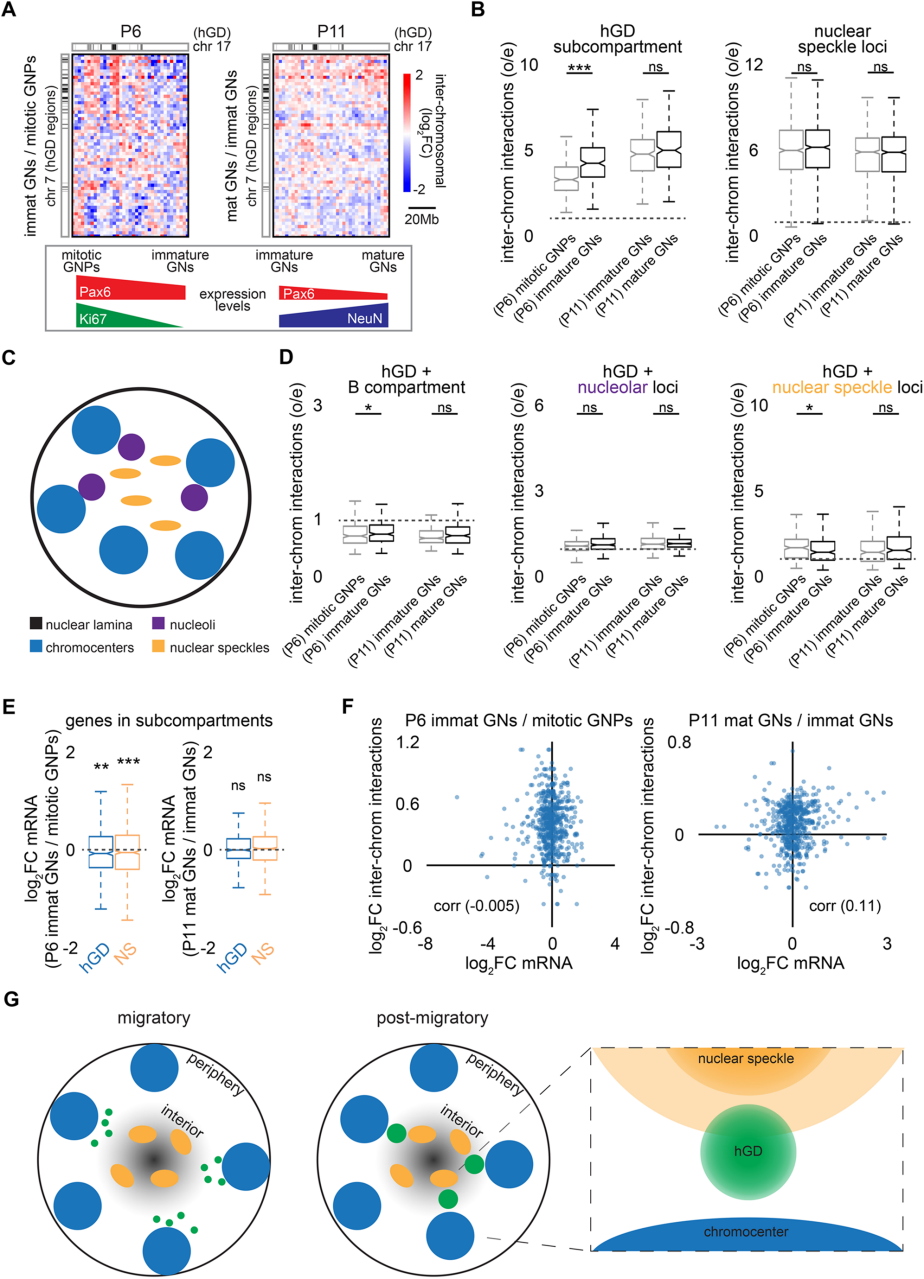
**Strengthening of the hGD subcompartment during early post-mitotic granule neuron development.** (A) FANS-isolated granule neuron populations from P6 or P11 mouse cerebellum as in Fig. 5B and F were subjected to Hi-C analyses. Fold changes in inter-chromosomal interactions between chromosome 7 and 17 are shown at 2.5Mb resolution, comparing the Ki67^high^/Pax6^high^ (mitotic GNPs) and Ki67^low^/Pax6^mid^ (immature GNs) populations from P6 mice (left), as well as the NeuN^mid^/Pax6^mid^ (immature GNs) and NeuN^high^/Pax6^mid-low^ (mature GNs) populations from P11 mice (right). (B) The inter-chromosomal interaction frequencies between regions within the hGD subcompartment or nuclear speckles across the various granule neuron populations indicated in A. Interactions between hGD regions significantly increased between mitotic granule precursors and immature post-mitotic granule neurons at P6, with little or no additional increase between immature and mature post-mitotic granule neurons at P11. (C) Schematic of nuclear bodies found in the nuclei of cerebellar granule neurons, including lamina-associated domains (dark blue), chromocenters (blue), nucleoli (purple), and nuclear speckles (yellow). (D) The inter-chromosomal interaction frequencies between hGD regions and B compartment loci (left), nucleolar-associated loci (middle), and nuclear speckle-associated loci (right). There were modest changes in inter-chromosomal interactions between hGD regions and other genomic regions associated with nuclear bodies across granule neuron development. (E) Fold changes in gene expression within hGD regions or nuclear speckle-associated loci across the various granule neuron populations indicated in A. Modest or no net changes in hGD or nuclear speckle-associated gene expression were observed across granule neuron development (**P<0.01, ***P<0.001 using asymptotic two-sample Brown-Mood median test, *n*=632, 1085, 586, 986 for P6 hGD, P6 NS, P11 hGD, P11 NS). (F) Pearson correlation between changes in gene expression within hGD regions and changes in their inter-chromosomal interactions with the hGD subcompartment across granule neuron populations indicated in A for P6 (left) or P11 (right) mice. Little or no correlations between changes in gene expression and changes in hGD subcompartment interactions were observed across development. (G) A model of the changes in nuclear organization observed during early cerebellar granule neuron development. hGD regions (green) are repositioned toward the nuclear interior and become more closely associated as immature neurons complete migration and progress to later developmental stages. In addition, hGD regions are developmentally relocated alongside their tethered chromocenters (blue) and may partly associate with nuclear speckles (orange) within the nuclear interior. In panels B,D, and E boxplots show median, first to third quartiles (boxes), and sample range (whiskers). Notches represent confidence intervals of the median. All data were collected from two independent biological replicates.

Having established an approach to profile granule neurons at different developmental stages, we employed high-throughput chromosome conformation capture (Hi-C) on FANS-isolated granule neuron populations to determine how the hGD subcompartment was reorganized during development (Fig. 6A). The Hi-C approach measures spatially proximal DNA-DNA interactions within a few hundred nanometers, making it suitable for characterizing the organization of genomic subcompartments, which may include interactions across chromosomes (Dekker et al., 2013; Finn et al., 2019). We observed a significant increase in inter-chromosomal interactions between regions within the hGD subcompartment when comparing Ki67^high^/Pax6^high^ granule neuron precursors with Ki67^low^/Pax6^mid^ immature post-mitotic granule neurons in the cerebellum of P6 mice (Fig. 6B, left). However, we observed little or no changes in inter-chromosomal interactions between hGD regions at later developmental stages when comparing NeuN^mid^/Pax6^mid^ immature granule neurons with NeuN^high^/Pax6^mid-low^ mature granule neurons in P11 mice. In contrast to the early developmental changes observed in the hGD subcompartment, we found that nuclear speckle-associated genomic loci (Quinodoz et al., 2018) maintained robust inter-chromosomal interactions throughout granule neuron development (Fig. 6B, right). These observations complement our DNA FISH analyses, which revealed that radial repositioning of a hGD region occurred as granule neurons became post-mitotic, and specifically after neuronal migration (Fig. S1B-E). These findings suggest that the strengthening of hGD subcompartment interactions and the nuclear relocalization of these regions occurred selectively during early post-mitotic granule neuron development.

Our imaging analyses had also revealed that hGD regions were physically tethered to heterochromatic chromocenters and exhibited modest association with transcriptionally active nuclear speckles (Fig. 4). We therefore next investigated how inter-chromosomal interactions between the hGD subcompartment and various other subcompartments might be developmentally changed. We observed that transcriptionally active hGD regions formed somewhat stronger inter-chromosomal interactions with nuclear speckle-associated loci compared to heterochromatic B compartment loci or nucleolar-associated loci and these differences persisted throughout development (Fig. 6C,D). Nevertheless, genomic interactions between hGD and nuclear speckle-associated loci were still lower compared to interactions between regions within each of these subcompartments (Fig. 6B,D), indicating that hGD regions and nuclear speckles remained as distinct transcriptionally active subcompartments. In addition, we found that inter-chromosomal interactions between hGD regions and genomic loci organized by nuclear bodies exhibited little overall changes during granule neuron development (Fig. 6D). These findings suggest that although microscopy revealed the coordinated repositioning of hGD regions and heterochromatic chromocenters toward the nuclear interior, hGD regions maintain a distinct genomic subcompartment that remains spatially separated from other nuclear structures throughout cerebellar granule neuron development.

Lastly, we investigated the relationship between the developmental reorganization of the hGD subcompartment and gene transcription. We found that genes residing within hGD regions and nuclear speckle-associated regions showed modest or no net changes in mRNA levels across granule neuron development (Fig. 6E). Moreover, changes in mRNA levels within hGD regions showed little or no correlation with changes in their inter-chromosomal subcompartment interactions across various stages of granule neuron development (Fig. 6F). These results suggest that the reorganization of the hGD subcompartment in developing granule neurons is not associated with gene transcription.

Altogether, we define the early developmental window in granule neurons during which hGD genomic regions are radially repositioned in the nucleus and their compartmentalization is strengthened, with these structural changes occurring independently of transcriptional changes in this subcompartment (Fig. 6G).

## DISCUSSION

Post-mitotic neurons undergo significant changes in the structural organization of their chromosomes in the developing brain. Conserved themes have emerged, where large genomic domains marked by heterochromatin show extensive repositioning away from the nuclear periphery during neuronal differentiation (Tan et al., 2021, 2023; Winick-Ng et al., 2021). In cerebellar granule neurons, we find that transcriptionally active gene-dense regions, located linearly adjacent to these heterochromatin domains, show coordinated radial repositioning together with heterochromatic chromocenters during development. These changes are associated with the overall strengthening of the active hGD subcompartment in developing granule neurons.

Interestingly, our results reveal that the structural reorganization of hGD regions occurred in immature granule neurons after they completed migration in the internal granule layer, as indicated by the downregulation of the microtubule-associated protein DCX. Prior to this, translocating nuclei are under frequent mechanical stress as they are pulled by cytoskeletal microtubules, thus requiring specific mechanisms to maintain nuclear structure during neuronal migration (Schreiner et al., 2015; Stephens et al., 2017; Tariq et al., 2017; Wu et al., 2018). Lamin and lamin receptor proteins are known to play roles in tethering heterochromatin to the nuclear lamina and in regulating nuclear stiffness during processes such as cell migration (Chen et al., 2019; Jung et al., 2013; Solovei et al., 2013). As post-mitotic neurons differentiate, lamin proteins may be downregulated, resulting in the detachment of heterochromatin regions from the nuclear lamina (Chang et al., 2022; Clowney et al., 2012; Harr et al., 2015; Solovei et al., 2013). Our finding that hGD regions are radially repositioned after the conclusion of granule neuron migration is consistent with the period when interactions between heterochromatic chromocenters and the nuclear lamina are weakened.

As hGD regions move away from the nuclear periphery in post-migratory granule neurons, they appear to settle in the nuclear interior, which is enriched for nuclear speckles. hGD regions share similar genomic features with nuclear speckle-associated regions, including a dense concentration of genes and enrichment for RNA polymerase II (Quinodoz et al., 2018; Zhao et al., 2023 preprint). However, the key difference is that hGD regions are linearly adjacent to large heterochromatin domains and are physically tethered to heterochromatic chromocenters in the nucleus. This likely limits the ability of transcriptionally active hGD regions to fully associate with nuclear speckles within a shared spatial compartment. Indeed, hGD regions were observed to maintain a partially separated compartment from other nuclear bodies, including nuclear speckles. Interestingly, we previously observed that the hGD subcompartment and nuclear speckles form higher-order genomic interactions using SPRITE, which captures interactions in the nuclear interior of granule neurons at spatial distances of up to a few micrometers (Quinodoz et al., 2018; Zhao et al., 2023 preprint). Because gene proximity to nuclear speckles is correlated with increased splicing of mRNA transcripts (Bhat et al., 2024), the higher-order genomic interactions between the hGD compartment and nuclear speckles may provide hGD regions with greater access to splicing factors concentrated within nuclear speckles. While our RNA-seq analyses failed to reveal overall changes in the transcription of genes within the hGD subcompartment during granule neuron development, it will be important in future studies to assess how other nuclear processes, including the splicing and nuclear export of transcripts, are regulated by structural changes in the hGD subcompartment.

In conclusion, our study reveals the specific stages of neuronal development during which chromosomes are reorganized. An active question remains as to what molecular mechanisms regulate these genome architectural changes. Besides the downregulation of nuclear lamina proteins, other genome architectural proteins, such as topoisomerases, may play a role by helping to resolve the entanglement of genomic DNA as chromosomes undergo significant reorganization, particularly following cell cycle exit (Hildebrand et al., 2024). Indeed, Top2b is abundantly expressed in post-mitotic neurons and plays key roles in their differentiation and function (Li et al., 2014; Segev et al., 2024; Tiwari et al., 2012). In addition to genome architectural proteins, chromatin remodeling enzymes such as Chd4 and Chd7 also have prominent functions in genome organization during granule neuron development (Goodman et al., 2020; Reddy et al., 2008). Elucidating how the modification of nucleosomes by these major chromatin enzymes influences chromosomal organization will provide valuable insights into how the genome is coordinately regulated across multiple length scales to drive granule neuron development.

## MATERIALS AND METHODS

### Animals

CD1 or C57BL/6 mice were purchased from Charles River Laboratories. Both male and female mice were used. All animal experiments were done according to protocols approved by the Animal Studies Committee of Northwestern University in accordance with the National Institutes of Health guidelines.

### Antibodies

Antibodies to DCX (Abcam, ab18723, lot# GR3441268-1), Ki67 (Abcam, ab15580, lot# GR3285096-1), NeuN (Abcam, ab190565, lot# 1001571-36 or ab279296), Nucleolin (Abcam, ab22758, lot# 1033948-1), Pax6 (Invitrogen, MA1-109), and SF3a66 (Abcam, ab77800) for immunohistochemistry or FANS experiments were purchased.

### Statistics

Statistical analyses were performed using R. Normality of data was assessed using the Shapiro–Wilk test, and homogeneity of variances was assessed using Bartlett's test. For experiments in two groups with non-normal distributions, the Mann–Whitney-Wilcoxon rank sum test (unpaired data) or the two-sample Brown-Mood median test (heterogeneously distributed data) was used. Comparisons of all samples across multiple groups were performed using the Kruskal–Wallis rank sum test followed by Dunn's post hoc testing with *P*-values adjusted using the Benjamini-Hochberg procedure for unpaired non-normal distributions.

### DNA FISH

DNA FISH assays were performed as described with modifications (Bolland et al., 2013; Yamada et al., 2019). The cerebellum of CD1 mice was fixed with 4% PFA and 4% sucrose by transcardiac perfusion. Sagittal cryosections (12 µm thickness) from 6-, 10-, 14-, and 22-day-old mice were prepared using a cryostat (Epredia) and cerebellar sections containing the vermis area were used for analyses. Sections were treated with sodium citrate solution (10 mM sodium citrate, pH 6.0) for 10 min at 70°C and cooled down to room temperature. Sections were dehydrated with sequential treatment of ice-cold 70% EtOH for 2 min, ice-cold 90% EtOH for 2 min, and ice-cold 100% EtOH for 2 min, and air-dried for 5 min. For hybridization, genomic DNA was denatured by incubating glass plates with buffer containing 2× SSC and 70% formamide for 2.5 min at 73°C, followed by incubation with 2× SSC and 50% formamide for 30 s.

All probes were made from BAC clones (BACPAC Resources Center) including RP24-97O16 (hGD1 locus), RP24-153C13 (hGD2 locus), RP24-97C4 (H1 locus), RP24-372C9 (H2 locus), and RP24-358P20 (NS1 locus) by nick translation with chemical coupling using an Alexa Fluor succinimidyl ester (Alexa 647 for hGD1 and NS1; Alexa 555 and Alexa 488 for hGD2, H1, and H2) (Thermo Fisher Scientific). The hybridization solution contained ∼70-80 ng of each labeled probe, 6 µg of mouse Cot-1 DNA (Thermo Fisher Scientific), and 10 µg of sheared salmon-sperm DNA (Thermo Fisher Scientific) in hybridization buffer (10% dextran sulfate, 50% formamide, 2xSSC). The probe mixtures were denatured at 73C for 5 min before use. Denatured sections and probes were sealed with coverslips and incubated at 37°C overnight. On the next day, coverslips were removed and washed once in 2xSSC and 50% formamide solution for 15 min at 45°C, two times in 2× SSC for 5 min at 45°C, and one time in 2xSSC for 5 min at room temperature with gentle agitation. Sections were washed once with 1xPBS and stained with the Hoechst 33342 DNA dye (Sigma, B2261). After washing with PBS, sections were mounted with Fluoromount-G mounting medium (Southern Biotech). Images were acquired on a confocal laser scanning microscope (Leica Microsystems, TCS SP8 confocal) using a 63× objective lens (NA 1.40). A stack of 0.3 µm thick optical sections was acquired for each field of view in the UV, red, and far-red channels. Two DNA FISH biological replicates were performed in all experiments.

#### Immunohistochemistry

Cerebellar sections were prepared and labeled with the relevant antibodies as previously described (Yamada et al., 2014). Secondary antibodies included anti-mouse or anti-rabbit Alexa 488 (Thermo Fisher Scientific, A11029 or A11034) or anti-mouse Alexa 647 (Thermo Fisher Scientific, A21235).

#### DNA FISH combined with immunohistochemistry

Cerebellar sections from 6-, 14-, and 22-day-old CD1 mice were prepared and treated with sodium citrate solution as described above. Sections were then labeled with the relevant primary and secondary antibodies. After extensive washing of secondary antibodies using PBS, sections were fixed again with 4% PFA in PBS for 10 min at room temperature, washed with PBS for 5 min twice, then 2xSSC twice for 5 min, and treated with ice-cold HCl solution (0.1 M HCl, 0.7% Triton-X) for 25 min at room temperature. Subsequently, DNA FISH procedures including prehybridization, hybridization, washing, and imaging were performed as described above. Two biological replicates were performed in all experiments.

#### DNA FISH and immunohistochemical analyses

For each cell, DNA FISH signals were subjected to automated identification using custom macros in ImageJ, as previously described (Xu et al., 2022; Yamada et al., 2019). The centroid of DNA FISH signals was detected using a 3D gaussian filter to reduce noise followed by 3D spot segmentation. For co-localization between pairs of DNA FISH probes, the Euclidian distance to the nearest alternate DNA FISH probe was calculated using the 3D Image Suite in ImageJ (Ollion et al., 2013).

Radial positioning of DNA FISH or immunofluorescence signals was analyzed using a single z-section in the middle third (z-axis) of a granule neuron nucleus to ensure a maximal x-y area for calculations. For DNA FISH analyses, the z-section containing the centroid of the DNA FISH probe signal was used. Loci failing to meet these criteria were excluded from analysis. For immunohistochemical analyses, the top 1% or top 10% pixels by intensity for each neuron were identified. The radial distances from the centroid of the nucleus to the DNA FISH probe signal or immunofluorescence signals were then calculated, where 0 represents the nuclear center and 1 represents the nuclear membrane.

The fluorescence intensity level surrounding a genomic locus was analyzed from the z-section containing the centroid of the DNA FISH probe signal using custom ImageJ macros. The average pixel intensity of the immunofluorescence signal in a 350 nm radius circle in the x-y plane surrounding the centroid was normalized to the bottom 20th and top 90th percentile of signal in the nucleus.

The borders of nuclear speckles were extracted as 3D objects using the top 2% pixels by intensity for each z-slice. The distance between the centroid of the DNA FISH probe signal and nearest nuclear speckle border was then quantified using the 3D Image Suite in ImageJ.

### FANS

The cerebellum from P6 or P11 C57BL/6 mouse pups was fixed with crosslinking solution (1% PFA in PBS) by homogenizing with a glass dounce homogenizer and incubated for 10 min at room temperature. The crosslinking reaction was quenched by 125 mM glycine solution and incubated for 5 min. The cell pellet was washed twice with BSA/PBS solution (0.3% BSA, 0.1% Triton-X in PBS) and stored at −80°C. To isolate nuclei, the frozen pellet was thawed on ice, homogenized with lysis buffer (10 mM Tris-HCl pH 8, 10 mM NaCl, 0.2% Triton-X) and incubated on ice for 10 min. The lysate was pelleted by centrifugation at 700 ***g*** for 5 min at 4°C and washed with lysis buffer once. The pellet was resuspended in BSA/PBS solution and filtered using a 50 µm filter (CellTrics). Filtered nuclei were incubated with various combinations of antibodies including anti-Pax6 (1/100), anti-Ki67 (1/100), or anti-NeuN (1/500) for 1 h at 4°C with rotation. Nuclei were washed with BSA/PBS solution twice and stained with secondary antibodies including anti-mouse Alexa 488 (1/250, Thermo Fisher Scientific, A11029) and anti-rabbit Alexa 647 (1/250, Abcam, ab150075) for 30 min at 4°C with rotation, followed by washing with BSA/PBS solution twice. Stained nuclei were resuspended in BSA/PBS solution and filtered using a 50 µm filter prior to FANS. The target populations were enriched with a SH800S cell sorter (Sony) using a 100 µm nozzle sorting chip and 488/638 nm lasers. Nuclei were first sorted using the ultra-yield (P6) or normal (P11) mode and then re-sorted using the normal mode.

#### RNA-seq

For RNA-seq following FANS, 1600-3500 cells were sorted and stored at −80°C. The sorted nuclei were thawed on ice and reverse-crosslinked with 100 µl of RIP buffer [100 mM Tris-HCl pH 8.0, 10 mM EDTA, 1% SDS, 1 µl of RNAse inhibitor (Promega)] containing 1 µl of Proteinase K (New England Biolab) for 1 h at 65°C. Then, nuclear RNA was purified using a RNeasy micro kit (Qiagen) according to the manufacturer's instructions. Half the amount of purified RNA from FANS-isolated granule neurons was treated with a NEBNext rRNA Depletion Kit (New England Biolabs). RNA-seq was performed using libraries prepared with a NEBNext Ultra™ Directional RNA Library Prep Kit for Illumina (New England Biolabs). RNA-seq libraries were sequenced on the Illumina NextSeq 550 platform to obtain 37 bp paired-end reads. Two biological RNA-seq replicates were performed in all experiments.

#### Hi-C

Hi-C assays were performed as previously described (Rao et al., 2014; Yamada et al., 2019). For Hi-C following FANS, 3500-4800 cells for P6 or 23,000-50,000 cells for P11 were sorted into PBS and lysis buffer (10 mM Tris-HCl pH 8, 10 mM NaCl, 0.2% Triton-X) at four times the volume was added immediately. Nuclei were pelleted and resuspended with 0.5% SDS for permeabilization as described with modifications (Rao et al., 2014; Yamada et al., 2019). Hi-C libraries were sequenced on the Illumina NextSeq 550 platform to obtain 37 bp paired-end reads. Two biological Hi-C replicates were performed in all experiments.

#### RNA-seq analyses

RNA-seq reads were aligned to the mm10 reference genome with HISAT2 using the public server at https://usegalaxy.org/ and normalized by library size. Gene annotations were derived using GENCODE version M11. Differential gene expression analyses were performed using pair-wise negative binomial tests with edgeR (Robinson et al., 2010) and the false discovery rate (FDR) was calculated for all genes.

snRNA-seq datasets from the developing cerebellum were obtained and analyzed using Seurat (Hao et al., 2021; Rosenberg et al., 2018). Briefly, granule neurons in the P11 developing cerebellum were extracted for downstream analysis. Read counts were normalized and variance stabilized using sctransform and subjected to dimensionality reduction using PCA preprocessing followed by UMAP embedding (Hao et al., 2021). Gene modules were obtained using differentially expressed genes from FANS-RNA-seq with log_2_FC>2 and FDR<0.05 for each granule neuron population. Genes that exhibited continuous upregulation or downregulation across all developmental stages, with an FDR<0.05 for each stage, were excluded from the immature granule neuron gene modules from P6 and P11 mice, which represent an intermediate developmental stage. To further distinguish the immature granule neuron modules from P6 and P11 mice, genes exhibiting higher expression in either P6 or P11 immature granule neurons were considered to represent the corresponding condition. Module scores were calculated from the average expression of genes within the module, subtracted by the levels of similarly expressed control genes (Hao et al., 2021).

#### ChIP-seq analyses

ChIP-seq datasets from P6 and P22 mouse cerebellum were obtained (Zhao et al., 2023 preprint). ChIP-seq reads were aligned to the mm10 reference genome with Bowtie2 using the public server at usegalaxy.org and normalized by library size. Normalized H3K9me3 counts were derived genome-wide at 500 Kb resolution and the differential ChIP-Seq signals (diffBind) between the P6 and P22 cerebellum were calculated using pair-wise negative binomial tests with EdgeR and the FDR. The frequency distribution was calculated using the fraction of bins with differential H3K9me3 signals per chromosome, normalized by the overall fraction of bins with differential signals across all chromosomes.

#### Hi-C and Dip-C analyses

Hi-C reads were aligned to the mm10 reference genome with Bowtie2 and HiC-Pro (Servant et al., 2015). Uniquely mapped paired-end reads were assigned to MboI restriction fragments and valid pairs with a minimum genomic distance of 1 Kb were filtered for PCR duplicates. Interaction matrices were normalized using Knight-Ruiz (KR) matrix balancing and observed divided by expected (o/e) transformation (Durand et al., 2016). Genomic regions associated with the hGD subcompartment, nuclear speckles, and nucleoli were obtained (Quinodoz et al., 2018; Zhao et al., 2023 preprint). A/B compartment strength was derived from the eigenvector of the Hi-C correlation matrix (Rao et al., 2014) using 500 Kb bins. Genomic loci enriched for the active histone modification H3K27ac and de-enriched for the repressive histone modification H3K9me3 were assigned a positive eigenvector and denoted as the active A compartment, while genomic loci with a negative eigenvector were denoted as the repressive B compartment.

Dip-C datasets from the developing and adult mouse cerebellum with cell type and structural stage annotations were obtained (Tan et al., 2023) and granule neurons were extracted for downstream analysis. Dimensionality reduction and visualization of Dip-C cells was performed using single-cell A/B compartment analysis as described (Tan et al., 2023), with modifications. Briefly, long-distance intra-chromosomal contacts separated by more than 3 Mb or inter-chromosomal contacts were kept for further analysis. Aligned reads were grouped into 100 Kb bins, and genomic bins with fewer than 10 long-distance contacts were excluded. For each bin, the average CpG density of contacting regions, weighted by the number of contacts, was calculated. A genomic bin x cell matrix was generated with each entry of the matrix containing the weighted CpG density of contacting regions. For bins with missing values, the weighted CpG density was interpolated from surrounding bins on the same chromosome, starting 1 bin away and extending to 2 bins if needed. Cells containing at least 5000 bins, as well as bins present in all cells, were considered for further analysis. The bin x cell matrix was then subjected to PCA preprocessing followed by UMAP embedding, as described for snRNA-seq analyses. Radial position analyses were performed from single cell 3D structures as previously described (Zhao et al., 2023 preprint), with modifications. Briefly, for 3D structures derived from F1 hybrid mice (Tan et al., 2023), the positions of genomic regions were projected onto the X-Y, Y-Z, or X-Z plane. Each plane is modeled as an ellipse based on the centroid and semi-axes calculated from datapoints in the plane. Planes with at least 40% of the maximum area among all planes in a stack were selected for downstream analysis. The 2D radial position was then calculated for each genomic region.

## Supplementary Material

10.1242/biolopen.062005_sup1 Supplementary information

Table S1. Genomic coordinates of hGD regions in mouse cerebellar granule neurons

Table S2. Differentially expressed transcripts between subpopulations of developing granule neurons
