# Supplementary material for: Reorganization of the heterochromatin-associated gene-dense subcompartment in early neuronal development
Source: Biol Open. 2025 May 12;14(5):bio062005. doi: 10.1242/bio.062005 (PMC12091228; doi:10.1242/bio.062005)
Supplement: Supplementary information [file biolopen-14-062005-s1.pdf]

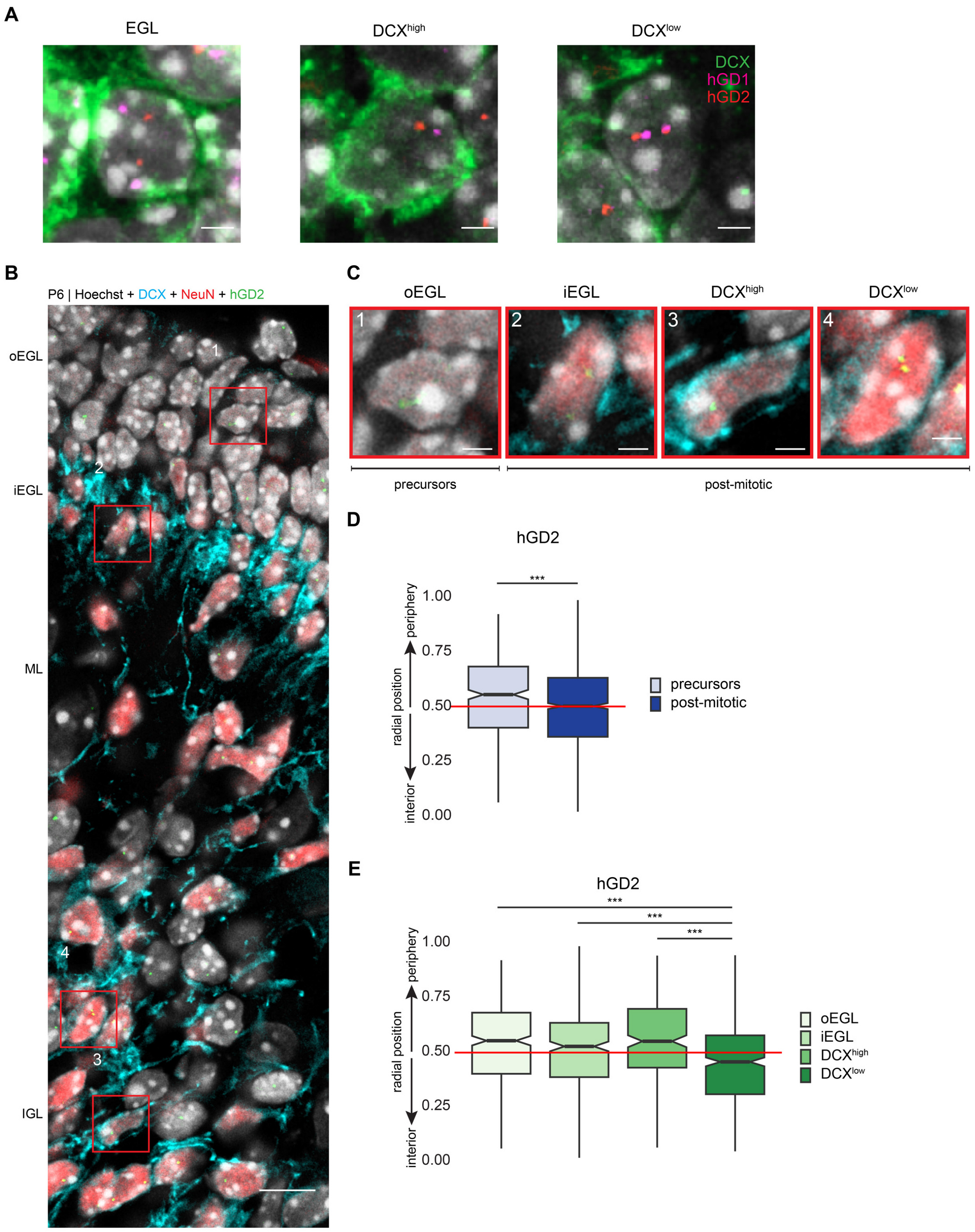

Biology Open • Supplementary information

Fig. S1. hGD loci are inwardly repositioned across post-mitotic neuron development

(A) Immunohistochemical analyses of cerebellar lobule 4/5 prepared from P14 mice as in Fig. 3C and labeled with Hoechst dye (gray), an antibody against DCX (green), and the hGD1 (pink) and hGD2 (red) DNA FISH probes. Scale bars: 2 µm. (B) Immunohistochemical analyses of cerebellar lobule 4/5 prepared from P6 mice and labeled with Hoechst dye (gray), antibodies against DCX (cyan) and NeuN (red), and the hGD2 (green) DNA FISH probe. Scale bar: 20 µm. (C) Four regions from B (red squares) depicting mitotic granule neuron precursors in the oEGL, immature post-mitotic granule neurons in the iEGL, and granule neurons in the IGL with either high DCX expression (DCX^{high}) or low DCX expression (DCX^{low}). Scale bars: 2 µm. (D) The radial positioning of hGD2 loci in granule neuron precursors or post-mitotic granule neurons, the latter marked by NeuN as shown in (C). hGD1 relocated closer toward the nuclear interior in post-mitotic granule neurons (*** $P < 0.001$ using Kruskal-Wallis rank sum test with Dunn's post-hoc test, n = 413, 993 for precursors, post-mitotic). (E) The radial positioning of the hGD2 locus in granule neurons across the four developmental stages shown in (C). hGD2 relocated to the nuclear interior in DCX^{low} post-migratory granule neurons (*** $P < 0.001$ using Kruskal-Wallis rank sum test with Dunn's post-hoc test, n = 413, 310, 242, 441 for hGD2 oEGL, iEGL, IGL DCX^{high}, IGL DCX^{low}). In panels D and E, boxplots show median, first to third quartiles (boxes), and sample range (whiskers). Notches represent confidence intervals of the median. All data was collected from two independent biological replicates.

Biology Open • Supplementary information

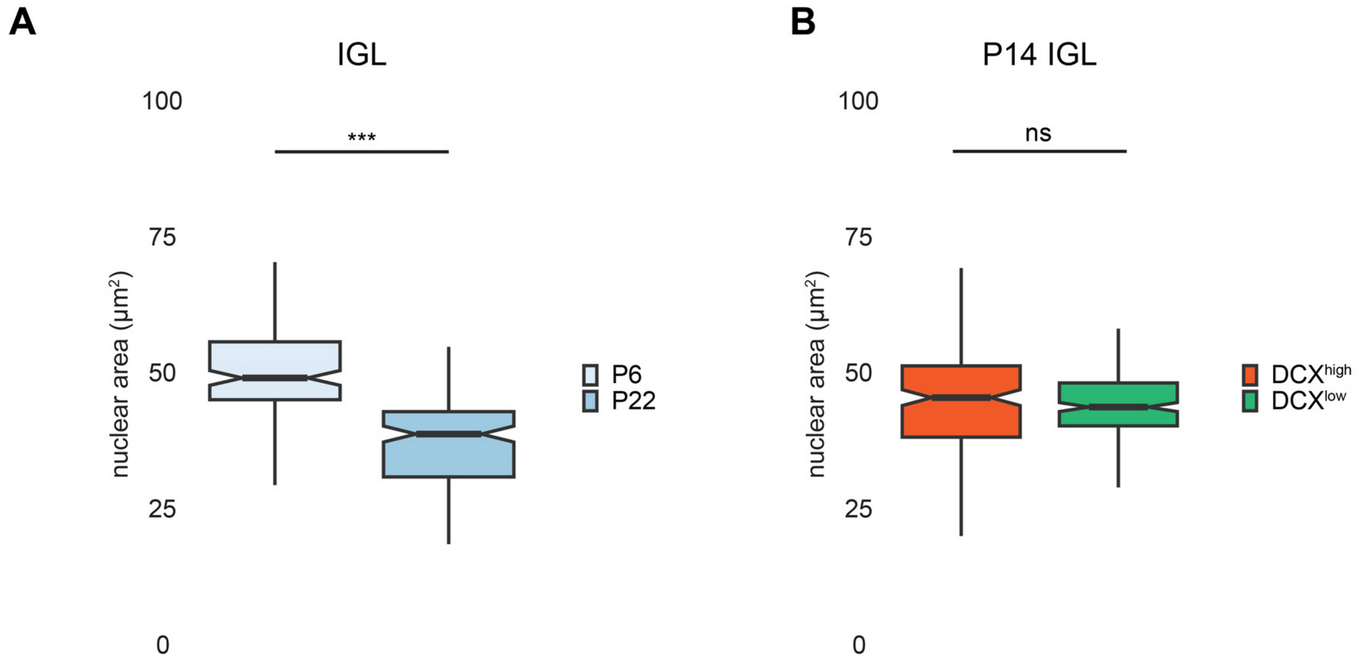

Fig. S2. Nuclear area varies with mouse age, but not with changes in DCX levels in granule neurons

(A) The nuclear area of post-mitotic granule neurons in the IGL of P6 and P22 mice. Granule neuron nuclear area decreased over the first three postnatal weeks (***$P < 0.001$ using two-sided Mann-Whitney-Wilcoxon rank sum test, n = 152, 166 for P6, P22). (B) The nuclear area of DCX^{high} and DCX^{low} granule neurons in the IGL of P14 mice. Granule neuron nuclear area exhibited little or no change with the downregulation of DCX ($P = 0.65$ using two-sided Mann-Whitney-Wilcoxon rank sum test, n = 200, 224 for DCX^{high}, DCX^{low}).

In panels A and B, boxplots show median, first to third quartiles (boxes), and sample range (whiskers). Notches represent confidence intervals of the median. All data was collected from two independent biological replicates.

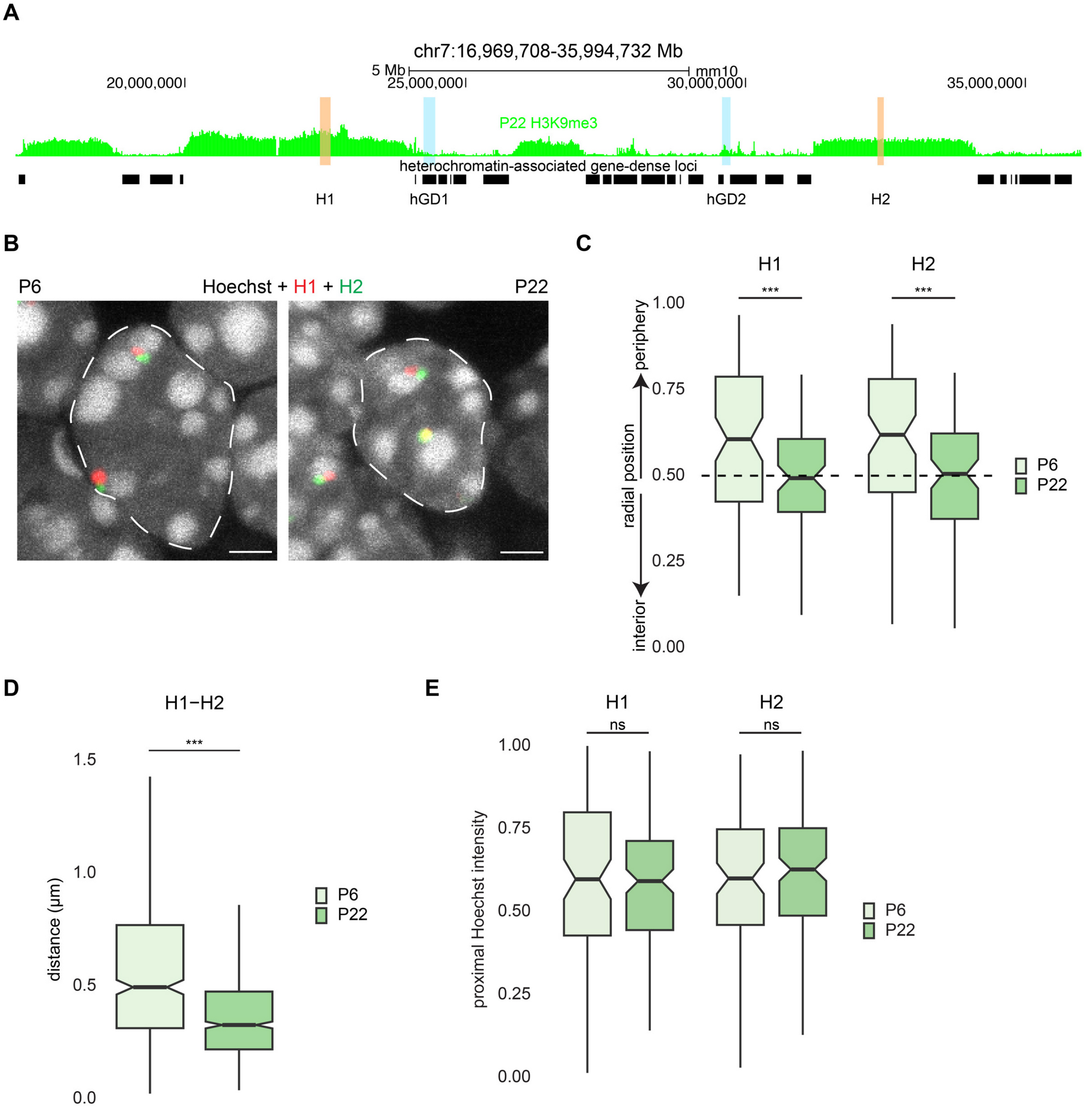

Biology Open • Supplementary information

Fig. S3. hGD proximal heterochromatin domains are inwardly repositioned during development

(A) UCSC genome browser track as shown in Fig. 1D, with the H1 and H2 regions, located within large heterochromatin domains adjacent to hGD regions, highlighted in orange. H1 is located 1.7Mb away from hGD1, and H2 is located 2.4Mb away from hGD2. (B) Granule neurons in the IGL of cerebellar lobule 4/5 from P6 (left) or P22 (right) mice subjected to DNA FISH analyses using the H1 (red) and H2 (green) probes, and stained with the Hoechst dye (gray). Dotted line marks the nuclear boundary. Scale bar: 2μm. (C) The radial positioning of H1 (left) and H2 loci (right) using the cerebellum from P6 and P22 mice as in Fig. 2D. Both heterochromatin loci were robustly repositioned toward the nuclear interior over cerebellar development (***$P < 0.001$ using two-sided Mann-Whitney-Wilcoxon rank sum test, n = 82, 83 for H1 P6, P22, n = 83, 84 for H2 P6, P22). (D) The 3D distances between the H1 and H2 probes in granule neurons from P6 and P22 mice prepared as in (B). The H1 and H2 loci increased their spatial proximity over cerebellar development (***$P < 0.001$ using two-sided Mann-Whitney-Wilcoxon rank sum test, n = 504, 701 for P6, P22). (E) Normalized Hoechst fluorescence intensity within a 350 nm radius circle surrounding the H1 and H2 loci in granule neurons from P6 or P22 cerebellum. Little or no changes in Hoechst fluorescence levels around the H1 and H2 heterochromatic regions were observed during cerebellar development ($P = 0.5349$, $P = 0.5078$ using Asymptotic Two-Sample Brown-Mood Median Test, n = 42, 65 for H1 P6, P22, n = 48, 65 for H2 P6, P22).

In panels C, D and E, boxplots show median, first to third quartiles (boxes), and sample range (whiskers). Notches represent confidence intervals of the median. All data was collected from two independent biological replicates.

Biology Open • Supplementary information

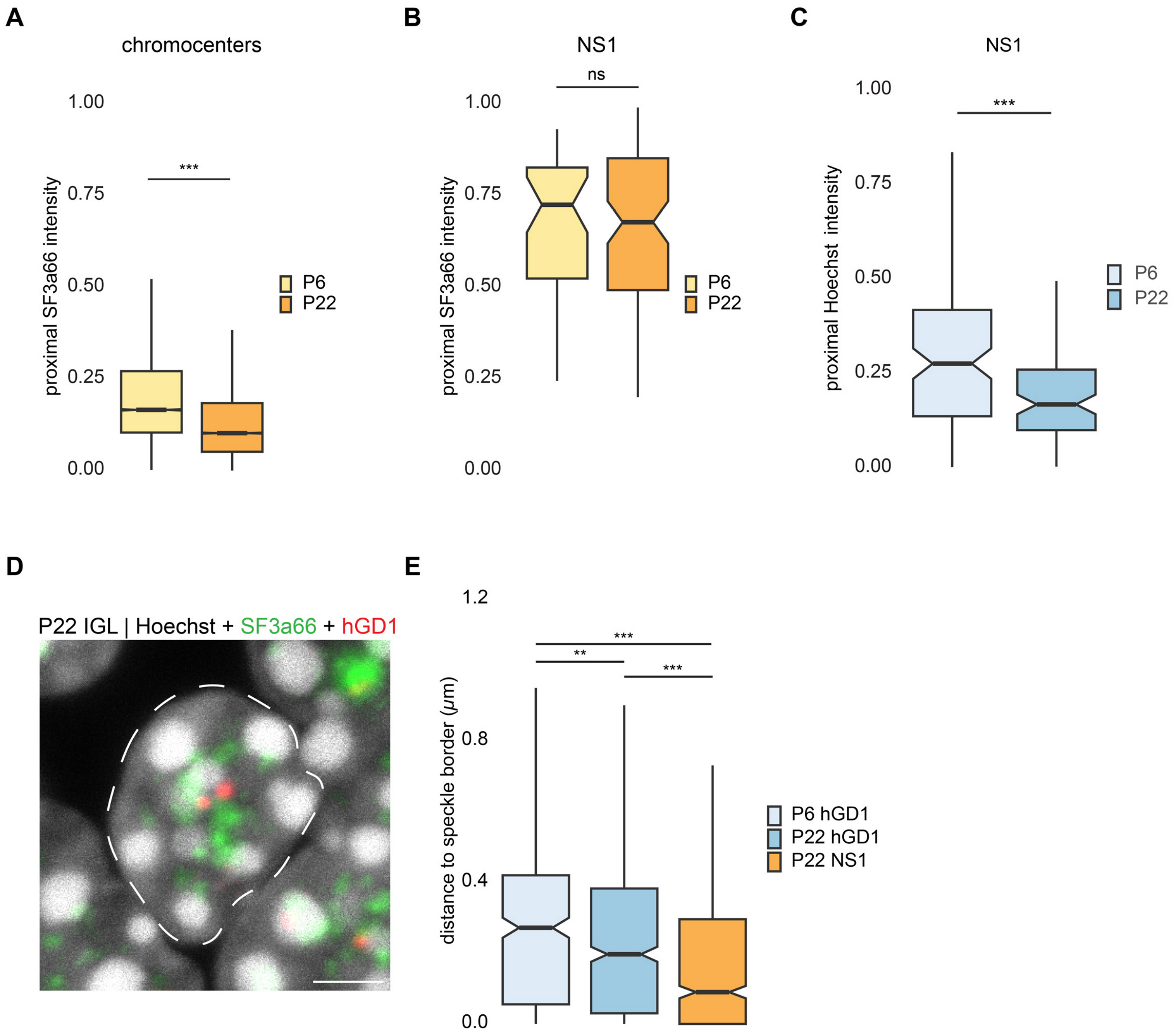
A
chromocenters
proximal SF3a66 intensity
1.00
0.75
0.50
0.25
0.00
***
P6
P22
B
NS1
ns
proximal SF3a66 intensity
1.00
0.75
0.50
0.25
0.00
P6
P22
C
NS1
***
proximal Hoechst intensity
1.00
0.75
0.50
0.25
0.00
P6
P22
D
P22 IGL | Hoechst + SF3a66 + hGD1
E
distance to speckle border (µm)
1.2
0.8
0.4
0.0
***
**
***
P6 hGD1
P22 hGD1
P22 NS1

Fig. S4. Localization of nuclear speckles relative to chromocenters and hGD regions in granule neurons

(A) Normalized SF3a66 fluorescence intensity within a 350 nm radius circle surrounding the top 10% of Hoechst signal intensity, representing chromocenters, within the nuclei of granule neurons from P6 or P22 cerebellum. Chromocenters exhibit reduced co-localization with SF3a66 during cerebellar development *(***P* < 0.001 using Asymptotic Two-Sample Brown-Mood Median Test, n = 22650, 33238 for P6, P22). (B) Normalized SF3a66 fluorescence intensity within a 350 nm radius surrounding the NS1 locus in granule neurons from P6 or P22 cerebellum. Little or no changes in SF3a66 fluorescence around the NS1 region were observed over development *(P* = 0.0518 using Asymptotic Two-Sample Brown-Mood Median Test, n = 88, 143 for P6, P22). (C) Normalized Hoechst fluorescence intensity analyzed as in (B) using P6 or P22 cerebellum. The NS1 locus showed reduced co-localization with Hoechst-enriched chromocenters during cerebellar development (****P* < 0.001 using Asymptotic Two-Sample Brown-Mood Median Test, n = 131, 139 for P6, P22). (D) Granule neurons in the IGL of cerebellar lobule 4/5 from P22 mice labeled with the hGD1 (red) DNA FISH probe, an antibody against SF3a66 (green), and Hoechst dye (gray). Dotted line marks the nuclear boundary. Scale bar: 2µm. (E) The 3D distances between the hGD1 centroid and the border of SF3a66-marked nuclear speckles as shown in (D). The hGD1 locus significantly decreased its distance to nuclear speckles over cerebellar development, but it remained significantly farther than NS1 (****P* < 0.001, ***P* < 0.01 using Kruskal-Wallis rank sum test with Dunn's post-hoc test, n = 418, 688, 704 for P6 hGD1, P22 hGD1, P22 NS1).

In panels A, B, C, and E, boxplots show median, first to third quartiles (boxes), and sample range (whiskers). Notches represent confidence intervals of the median. All data was collected from two independent biological replicates.

Biology Open • Supplementary information

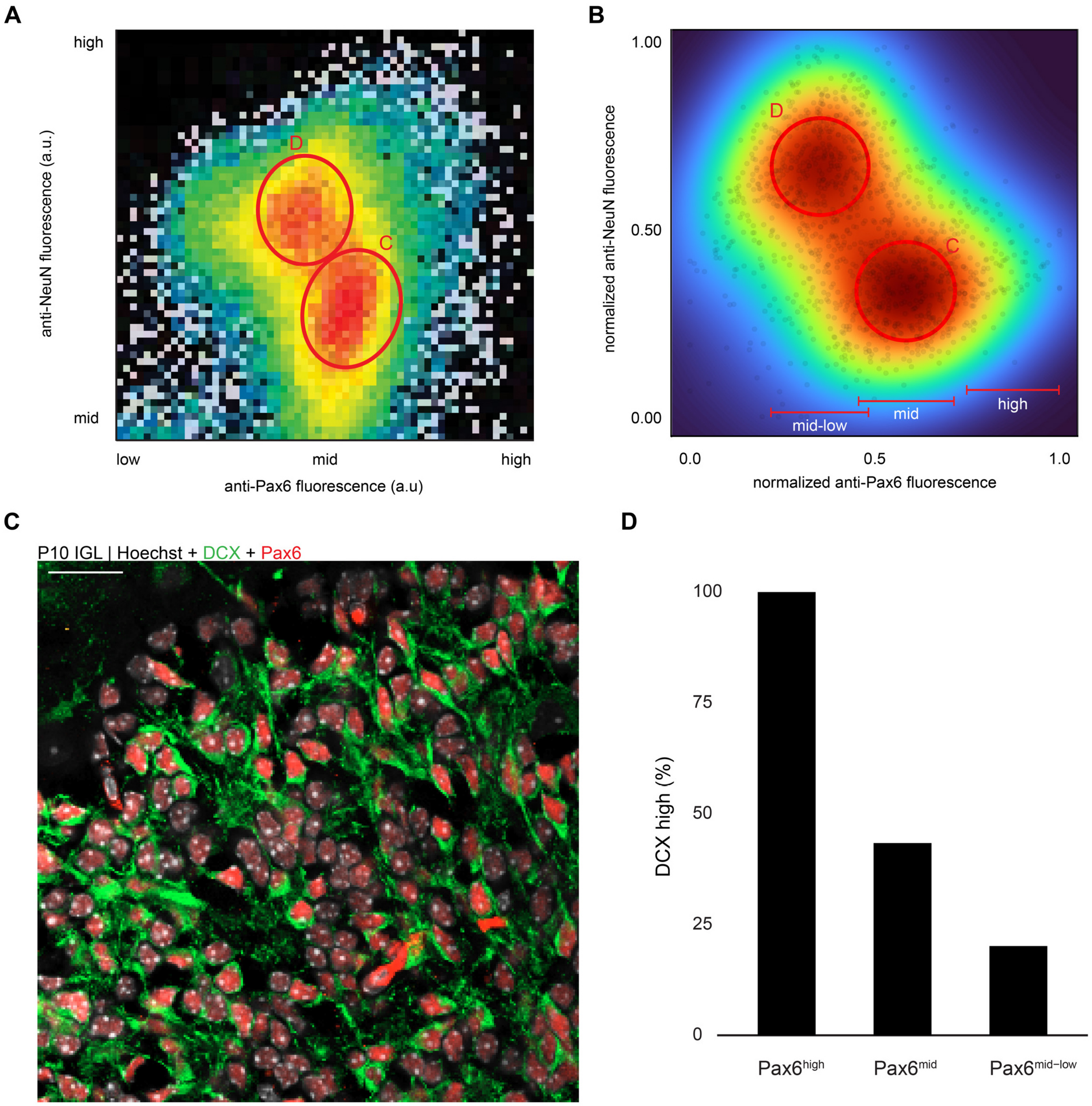

Biology Open • Supplementary information

Fig. S5. The Pax6^{high} granule neuron subpopulation is enriched for DCX^{high} migratory neurons

(A) Granule neurons from P11 mice labeled with the Pax6 and NeuN antibodies and subjected to FANS as in Fig. 5F. Groups D and C represent NeuN^{high}/Pax6$^{mid-low}$ and NeuN^{mid}/Pax6^{mid} granule neuron populations, respectively. (B) Density heat map plot of Pax6 and NeuN immunofluorescence in granule neurons from P10 mice as shown in Fig. 5E. Individual neurons are marked in gray, while groups D and C represent NeuN^{high}/Pax6$^{mid-low}$ and NeuN^{mid}/Pax6^{mid} granule neuron populations, respectively. Pax6^{high} granule neurons are indicated, but represent a relatively small population in P10 mice. (C) Immunohistochemical analyses of cerebellar lobule 4/5 prepared from P10 mice labeled with Hoechst dye (gray), together with antibodies against DCX (green) and Pax6 (red). Scale bar: 20 µm. (D) The fraction of Pax6^{high}, Pax6^{mid}, or Pax6$^{mid-low}$ granule neurons, stratified by Pax6 levels as in (B), containing high DCX expression (DCX^{high}). Migratory granule neurons, characterized by high DCX expression, primarily overlapped with Pax6^{high} granule neurons.

Biology Open • Supplementary information

Table S1. Genomic coordinates of hGD regions in mouse cerebellar granule neurons

Available for download at
https://journals.biologists.com/bio/article-lookup/doi/10.1242/bio.062005#supplementary-data

Table S2. Differentially expressed transcripts between subpopulations of developing granule neurons

Available for download at
https://journals.biologists.com/bio/article-lookup/doi/10.1242/bio.062005#supplementary-data
